# The CXCR4–STAT3–IL-10 Pathway Controls the Immunoregulatory Function of Chronic Lymphocytic Leukemia and Is Modulated by Lenalidomide

**DOI:** 10.3389/fimmu.2017.01773

**Published:** 2018-01-15

**Authors:** Hila Shaim, Zeev Estrov, David Harris, Mayra Hernandez Sanabria, Zhiming Liu, Peter Ruvolo, Phillip A. Thompson, Alessandra Ferrajoli, May Daher, Jan Burger, Muharrem Muftuoglu, Nobuhiko Imahashi, Li Li, Enli Liu, Abdullah Saleh Alsuliman, Rafet Basar, Lucila Nassif Kerbauy, Catherine Sobieski, Elif Gokdemir, Kayo Kondo, William Wierda, Michael Keating, Elizabeth J. Shpall, Katayoun Rezvani

**Affiliations:** ^1^Department of Stem Cell Transplantation and Cellular Therapy, The University of Texas MD Anderson Cancer Center, Houston, TX, United States; ^2^Department of Leukemia, The University of Texas MD Anderson Cancer Center, Houston, TX, United States

**Keywords:** chronic lymphocytic leukemia, B10, regulatory B cells, immunosuppression, STAT3, IL-10, CXC chemokine ligand 12/CXCR4 axis, lenalidomide

## Abstract

Chronic lymphocytic leukemia (CLL) cells possess regulatory functions comparable to those of normal B10 cells, a regulatory B cell subset that suppresses effector T-cell function through STAT3-mediated IL-10 production. However, the mechanisms governing IL-10 production by CLL cells are not fully understood. Here, we show that the CXC chemokine ligand 12 (CXCL12)–CXCR4–STAT3 axis regulates IL-10 production by CLL cells and their ability to suppress T-cell effector function through an IL-10 mediated mechanism. Knockdown of STAT3 significantly impaired the ability of CLL cells to produce IL-10. Furthermore, experiments to assess the role of lenalidomide, an immunomodulatory agent with direct antitumor effect as well as pleiotropic activity on the immune system, showed that this agent prevents a CXCL12-induced increase in p-S727-STAT3 and the IL-10 response by CLL cells. Lenalidomide also suppressed IL-10-induced Y705-STAT3 phosphorylation in healthy T cells, thus reversing CLL-induced T-cell dysfunction. We conclude that the capacity of CLL cells to produce IL-10 is mediated by the CXCL12–CXCR4–STAT3 pathway and likely contributes to immunodeficiency in patients. Lenalidomide appears to be able to reverse CLL-induced immunosuppression through including abrogation of the CXCL12–CXCR4–S727–STAT3-mediated IL-10 response by CLL cells and prevention of IL-10-induced phosphorylation of Y705-STAT3 in T cells.

## Introduction

Chronic lymphocytic leukemia (CLL) is characterized by generalized immune suppression and susceptibility to infectious complications and secondary malignancies (1–3). Although a number of studies have reported both quantitative and qualitative defects in T-cell function in CLL (4, 5), the mechanisms underlying CLL-induced immunosuppression have been difficult to dissect, as they involve complex bidirectional interactions among leukemic cells, components of the tumor microenvironment and immune effectors (6–10).

A subset of B cells known as regulatory B cells (Bregs) have been shown to contribute to the maintenance of tolerance, primarily *via* STAT3-mediated production of IL-10 (known as B10 cells) in both mice (11, 12) and humans (13–16). B10 cells have been implicated in the pathogenesis of autoimmune disorders, such as systemic lupus erythematosus, allergic dermatitis, multiple sclerosis, as well as alloimmune disorders such as graft-versus-host disease (12, 13, 17–20). DiLillo et al. (21) recently reported that CLL cells are capable of secreting IL-10 and possess regulatory functions comparable to those of normal B10 cells. Moreover, IL-10 is elevated in the serum of CLL patients (22). These intriguing observations suggest a means by which CLL cells could induce immunosuppression in patients; but a mechanistic basis for IL-10 production by CLL cells is still lacking.

The CLL microenvironment supports tumor cell survival *via* secretion of a number of soluble and surface-bound factors, including CXC chemokine ligand 12 (CXCL12) (6, 9). CXCL12 binds its receptor CXCR4 on the surface of CLL cells and directs chemotaxis, supports tumor survival, and activates various signaling pathways, including STAT3 (6, 9, 23).

Here, we report that the capacity of CLL to produce IL-10 is regulated by the CXCL12–CXCR4–STAT3 pathway and may contribute to immunodeficiency in patients. Treatment with the immunomodulatory agent lenalidomide prevented IL-10 production by CLL cells, as well as IL-10-induced T-cell dysfunction, by inhibiting activation of the STAT3 pathway. Our data provide a novel mechanism for T-cell dysfunction in CLL, involving the CXCL12–CXCR4–STAT3 signaling pathway and CLL B10 function, and provide additional targets of lenalidomide that can account for its therapeutic immunomodulatory effect in CLL.

## Materials and Methods

### Patients

Twenty-six patients with CLL (Table 1) were recruited from the University of Texas MD Anderson Cancer Center (MDACC). None had received therapy for at least 2 years or more and all gave written informed consent according to protocols approved by the MDACC institutional review board. Peripheral blood mononuclear cells (PBMCs) were purified with Lymphoprep for density gradient separation. Cells were cryopreserved in freezing media containing 90% FBS (fetal bovine serum) and 10% DMSO and stored in liquid nitrogen.

**Table 1:** Chronic lymphocytic leukemia patient characteristics.

Median age (range)	65 years (44–76)
**Gender:**	
Female	12 (46%)
Male	14 (54%)
**Rai stage:**	
0	13 (50%)
1	4 (15%)
2	0
3	4 (15%)
4	5 (20%)
**Zap70:**	
Zap70−	12 (42%)
Zap70+	11 (46%)
Unknown	3 (12%)
**IgV_H_ mutation status:**
IgH_H_−	9 (34.5%)
IgV_H_+	15 (58%)
Unknown	2 (7.5%)
**Cytogenetics:**	
13q	16 (61.5%)
t12	3 (11.5%)
TP53	5 (19%)
No mutations	1 (4%)
Unknown	1 (4%)
**Secondary malignancies:**	
Yes	4 (15%)
No	22 (85%)
Previous therapy	1 (4%)
No previous therapy	25 (96%)

### Characterization of IL10+ CD19+ CD5+ CLL Cells

To characterize IL-10+ B cell, we thawed cryopreserved PBMCs from CLL patients and healthy controls and stimulated them with CpG (4 µg/ml) (positive control) or CXCL12 (250 ng/ml) (R&D) for 14–16 h. Phorbol myristate acetate (PMA; 50 ng/mL), ionomycin (250 ng/mL, Sigma Aldrich) and brefeldin A (10 µg/mL; Sigma-Aldrich) were added for the last 6 h of culture. Cells were harvested, washed, and incubated for 20 min at room temperature with a cocktail of anti-CD19-PE-cy7, CD5-FITC (BioLegend), and Live/Dead-Aqua (Invitrogen). Cells were then washed and fixed/permeabilized for 1 h at 4°C with foxp3 staining buffer (eBioscience) according to the manufacturer’s instructions. Cells were incubated for 30 min at room temperature with rat APC-conjugated anti-IL-10 or rat IgG2aК isotype antibodies (BD Biosciences). For blocking experiments, we performed the assay in the presence or absence of anti-CXCR4 blocking antibody (BioLegend), the STAT3 inhibitor cucurbitacin (0.05 µM) or lenalidomide (10 µM). To determine apoptosis, cells were stained with annexin V-FITC and 7-AAD using the Annexin V Apoptosis Detection Kit (BD Biosciences) according to the manufacturer’s instructions. All data were acquired with BD LSRFortessa (BD Biosciences) and analyzed with FlowJo software version 10.

### Phosflow Assay

To analyze S727-STAT3 phosphorylation in CLL cells, we cultured PBMCs from CLL patients in the presence or absence of the STAT3 inhibitor cucurbitacin (0.05 µM) for 2 h or lenalidomide (10 µM) for 2 or 24 h at 37^o^C. Cells were stained for 30 min with Live/Dead-Aqua (Invitrogen). Samples were stimulated with 250 ng/ml of CXCL12 for 20 min at 37^o^C, and then fixed for 10 min in the dark. After one wash and 20 min of surface staining with CD19-APC (BD Biosciences) and CD5-FITC (BioLegend) antibodies, the cells were washed, permeabilized (Phospho-Epitopes Exposure kit-Beckman Coulter kit), and stained with p-S727-STAT3-PE mAb Phosflow antibody (BD Biosciences) for 30 min at room temperature.

To analyze Y705-STAT3 phosphorylation in T cells, we incubated healthy donor PBMC for 20 min at 37^°^C with IL-10 (10 ng/ml) (R&D) or with supernatants collected from CXCL12-treated CLL cells (supernatant was harvested after CLL cells were treated with 250 ng/ml of CXCL12 for 48 h). Cells were fixed and stained with anti-CD3-v450 (BD Biosciences) and phosphorylated Y705-STAT3-PE mAb Phosflow antibody (BD Biosciences), following the protocol described above. To examine the effect of lenalidomide on Y705-STAT3 phosphorylation in T cells we pre-incubated the T cells for 2 h with lenalidomide (10 µM) before adding IL-10/CLL cells supernatant.

### T Cell Suppression Assay

T cells were magnetically purified from PBMC using a PAN T-cell isolation kit (Miltenyi Biotec) from healthy donors. Healthy donor T cells (1 × 10^5^) were then cultured with bead-purified (Miltenyi Biotec) CD19+ B cells from CLL patients (1 × 10^6^) at an optimal 1:10 ratio per well in the presence or absence of CXCL12 (250 ng/ml), lenalidomide (10 µM), CXCR4, or mouse anti-human IL-10 blocking antibodies (2 µg/ml, R&D). Each experiment was performed in duplicate. After 48 h of culture, T cells were magnetically purified again as described above (purity >95%) and activated with anti-CD3/CD28 magnetic Dynabeads (Invitrogen) for 6 h. A negative control (no stimulation) was included in every experiment. Brefeldin A (10 µg/mL) and CD107a PE-CF594 (BD Biosciences) were added to the culture. Cells were stained with a Live/Dead-Aqua (Invitrogen), CD3-BV650, CD8-FITC, CD4-APC-Cy7, fixed/permeabilized (BD Biosciences) followed by intracellular staining with IFN-γ-V450, TNF-α-Alexa 700, and IL-2-PE (BD Biosciences).

### Proliferation Assay

Magnetically purified carboxyfluorescein succinimidyl ester-labeled (Invitrogen) healthy donors T cells were (Invitrogen) cultured with CLL cells at an optimal 1:10 ratio per well in the presence or absence of CXCL12 (250 ng/ml) and activated with anti-CD3/CD28 Dynabeads. After 3 days, the cells were collected; stained with Live/Dead-Aqua (Invitrogen), CD3-BV650, and CD19-PE-cy7; and analyzed by flow cytometry.

### Western Blotting

Peripheral blood mononuclear cells from CLL patients were cultured in the presence or absence of 10 µM lenalidomide for 2 h and then stimulated with or without 250 ng/ml of CXCL12 for 20 min at 37°C. Cell pellets were lysed using 1 × Pierce IP Lysis Buffer together with 1 × Halt Protease and Phosphatase Inhibitor Cocktail (Thermo-Fisher-Scientific). Protein from each sample (20 µg) was then loaded on NuPAGE 4–12% Bis-Tris Protein gel and separated using NuPAGE MOPS SDS running buffer (Thermo-Fisher-Scientific). The proteins was transferred to a nitrocellulose membrane in NuPAGE Transfer Buffer plus 10% methanol and stained with mouse anti-human monoclonal antibodies against STAT3 and β-actin, and polyclonal rabbit anti-human p-S727-STAT3 (Cell Signaling). IRDye 680RD goat anti-mouse and 800CW goat anti-rabbit antibodies (Li-Cor) were used as secondary antibodies. Blots were visualized using Odyssey Imaging System (Li-Cor). Densitometry values were normalized using the values of the loading control (actin from untreated CLL was used as reference). The numerical value for the signal of p-S727-STAT3 from each sample was divided by the numerical value of the signal from the corresponding total STAT3.

### Generation of Green Fluorescence Protein (GFP)-Lentiviral STAT3-Short Hairpin RNA (shRNA) and Infection of CLL Cells

293T cells were co-transfected with GFP-lentivirus STAT3 shRNA or GFP-lentivirus empty vector and the packaging vectors pCMVdeltaR8.2 and pMDG, using the Superfect transfection reagent (Qiagen, Inc.). The 293T cell culture medium was changed after 16 h, collected after 48 h, and filtered through a 45-µm syringe filter to remove floating cells. The lentivirus was then concentrated by filtration through an Amicon ultracentrifugal filter device (Millipore), and the concentrated supernatant was used to infect CLL cells. CLL cells from three different patients (5 × 10^6^ cells per experiment) were incubated in DMEM supplemented with 10% fetal calf serum and transfected with viral supernatant as described by Rozovski et al. (24) The transfection rate for the empty vector was 23.9% (range 19.5–27%) and for the STAT3 vector 21% (range 18.5–21.5%).

### Statistical Analyses

All values are reported as means and SEM. Statistical significance was assessed with the Prism 5.0 software (GraphPad Software, Inc.), using unpaired or paired two-tailed *t*-tests as appropriate. A *P* ≤ 0.05 was considered to indicate statistical significance. To standardize comparisons of median fluorescence intensities (MFI), we used a resolution index (MFI index) (*X*pos − *X*negative)/√(SDpos^2^ + SDnegative^2^) (25, 26).

## Results

### CXCL12–CXCR4 Interaction Induces Phosphorylation of S727-STAT3 and IL-10 Production by CLL Cells

**Figure 1:**
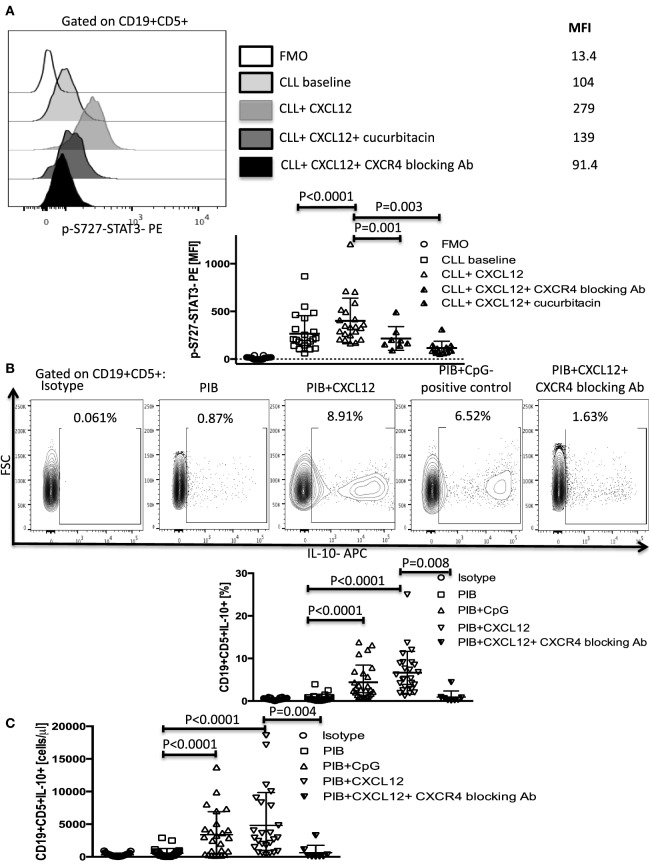
CXC chemokine ligand 12 (CXCL12)–CXCR4 interaction induces phosphorylation of S727-STAT3 and IL-10 production by chronic lymphocytic leukemia (CLL) cells. **(A)** CXCL12 phosphorylates S727-STAT3 in CLL cells through interaction with the CXCR4 receptor. CLL cells were stimulated with 250 ng/ml CXCL12 for 20 min, fixed, permeabilized, and stained for p-S727-STAT3. Exposure to 0.05 µM/ml of the STAT3 inhibitor cucurbitacin for 2 h or CXCR4 blocking antibody, prevented the CXCL12-induced S727-STAT3 phosphorylation (*n* = 19). **(B,C)** Cytoplasmic IL-10 expression by CLL cells after CXCL12 stimulation. CLL cells were cultured for 8–10 h with either 250 ng/ml CXC12 or 4 µg/ml CpG (positive control), followed by addition of Phorbol myristate acetate (PMA), ionomycin, and BFA (PIB), and then incubated for another 6 h before performing intracellular staining. CXCL12 induced an increase in IL-10+ CLL cells, both in frequencies **(B)** and in absolute counts **(C)**.

The chemokine CXCL12 is constitutively secreted by bone marrow stroma cells in CLL and binds CXCR4 on the surface of CLL cells to direct chemotaxis, support tumor survival, and activate various signaling pathways, including STAT3 by phosphorylation of its serine 727 residue (6, 9). CXCL12 induction of STAT3 phosphorylation has also been reported in other types of cancers, such as bladder cancer (27), breast cancer (28, 29), and small cell lung cancer (30). However, in CLL, phosphorylation of the S727-residue rather than the Y705 has been shown to be important for the tumor-promoting function of STAT3 (31). To determine if CXCL12 can promote IL-10 production in CLL cells by activating the STAT3 pathway, we stimulated CLL cells with 250 ng/ml of CXCL12 and measured p-S727-STAT3 as described in the Section “Materials and Methods.” CXCL12 induced a significant increase in the levels of p-S727-STAT3 (Figure 1A), but not Y705-STAT3 (Figure S1 in Supplementary Material) in CLL cells. This effect was abrogated when the cells were cultured in the presence of a CXCR4 blocking antibody or a STAT3 inhibitor (cucurbitacin) (Figure 1A). Cucurbitacin is a highly selective inhibitor of STAT3 signaling (32, 33) reported to target STAT3 in CLL (33), with no significant effect on other pro-survival pathways within malignant cells, such as Ras–raf–MEK–ERK and PI3k/Akt. Notably, overnight culture of CLL cells with 0.05 µM cucurbitacin did not significantly affect cell viability or the levels of total STAT3 (Figures S2A,B and S3 in Supplementary Material).

We next asked if activation of the p-S727-STAT3 pathway by CXCL12–CXCR4 interaction results in IL-10 production in CLL cells. Using CLL cells from 26 patients and 5 healthy controls, we found that both CXCL12 (mean 6.9 ± 1% SEM, *P* < 0.0001) and CpG (positive control) (4.6 ± 0.8%, *P* < 0.0001) induced significant IL-10 production by CLL cells, as measured by intracellular staining and ELISA (Figures 1B,C; Figure S4A in Supplementary Material). CpG also induced phosphorylation of the S727 residue of STAT3 in CLL cells (Figure S4B in Supplementary Material). By contrast, whereas healthy peripheral blood B cells produced IL-10 following stimulation with 4 µg/ml CpG, they failed to respond to CXCL12, pointing to a unique role for the CXCL12–CXCR4 axis in mediating IL-10 response in CLL cells (Figure S4C in Supplementary Material). The addition of a CXCR4 blocking antibody abolished the CLL IL-10 response to CXCL12, further confirming the notion that the CXCL12–CXCR4 axis is critical for IL-10 production by B-CLL cells. We also found that CXCR4 expression on the surface of CLL cells (based on standardized MFI index) positively correlates with their ability to secrete IL-10 in response to CXCL12 stimulation (Figure S5 in Supplementary Material), suggesting that CXCR4 expression could serve as a predictor of CXCL12-induced IL-10 response in CLL.

**Figure 2:**
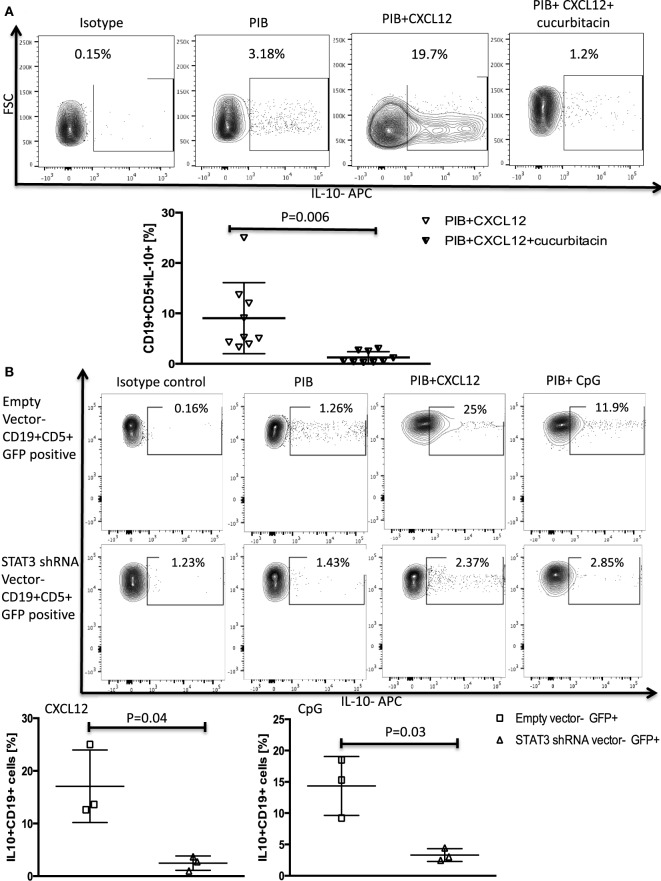
STAT3 inhibition abrogates CXC chemokine ligand 12 (CXCL12)–CXCR4 induced IL-10 production in chronic lymphocytic leukemia (CLL) cells. **(A)** The STAT3 inhibitor cucurbitacin inhibits CXCL12-induced IL-10 production by CLL cells. CLL cells were primed with 0.05 µM of the STAT3 inhibitor cucurbitacin for 2 h, and then stimulated with CXCL12 and phorbol myristate acetate (PMA), ionomycin and BFA (PIB) (*n* = 9). **(B)** STAT3 knockdown using a green fluorescence protein (GFP)-lentivirus STAT3 short hairpin RNA (shRNA) resulted in abrogation of CXCL12 and CpG-induced IL-10 production in GFP+ CLL cells compared to CLL cells transfected with the empty vector (*n* = 3).

We confirmed the involvement of STAT3 as a mediator of CXCL12–CXCR4-induced IL-10 production by both incubating CLL cells with cucurbitacin for 2 h before adding CXCL12 and by knocking down STAT3 by shRNA as described in the Section “Materials and Methods.” These modifications led to near complete abrogation of IL-10 production by B-CLL cells (Figures 2A,B) without any significant impact on CLL viability (Figures S2B–D in Supplementary Material).

Taken together, these data support a leading role for the CXCL12–CXCR4–STAT3 pathway in mediating IL-10 production by B-CLL cells.

### CXCL12-Mediated IL-10 Production by CLL Cells Induces T-Cell Suppression through Phosphorylation of Y705-STAT3

**Figure 3:**
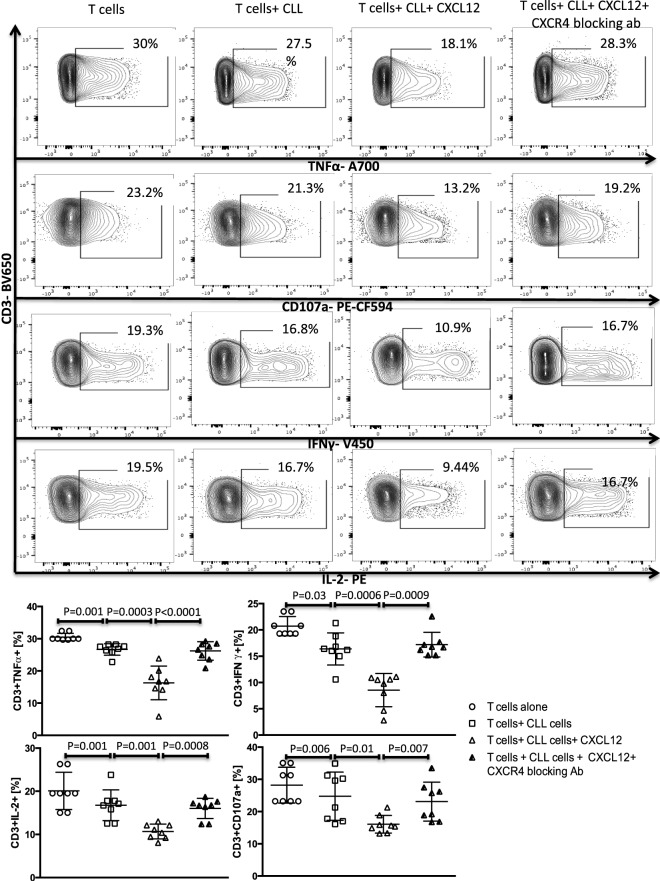
The CXC chemokine ligand 12 (CXCL12)–CXCR4 axis mediates chronic lymphocytic leukemia (CLL)-induced T-cell suppression. T cells were cocultured with CLL cells at a 1:10 ratio for 48 h, with or without 250 ng/ml CXCL12. The T cells were then isolated and stimulated with anti-CD3/CD28 beads for 6 h and stained for IFNγ, TNFα, IL-2, and CD107a. The addition of CXCL12 to the coculture induced T cell dysfunction by the CLL cells. Blocking mAbs to CXCR4 inhibited CXCL12–CLL-induced suppression of T cells (*n* = 6). FACS plots are gated on live CD3+ cells.

**Figure 4:**
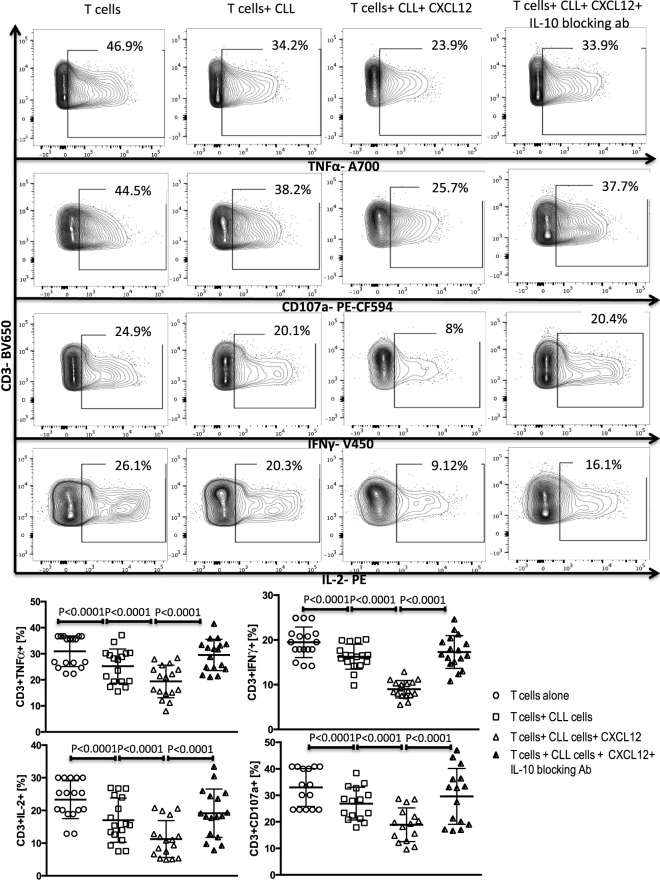
CXC chemokine ligand 12 (CXCL12) mediates CLL-induced T-cell suppression through IL-10 production by the CLL cells. T cells were cocultured with CLL cells at a 1:10 ratio for 48 h, with or without 250 ng/ml CXCL12. The T cells were then isolated and stimulated with anti-CD3/CD28 beads for 6 h and stained for IFNγ, TNFα, IL-2, and CD107a. The addition of CXCL12 to the coculture induced T cell dysfunction by the CLL cells. Blocking or IL-10 inhibited CXCL12–CLL-induced suppression of T cells (*n* = 17). FACS plots are gated on live CD3+ cells.

A subset of Bregs has been functionally defined in humans and mice by their ability to express IL-10 (11, 14). This capacity is central to the ability of these B10 cells to negatively regulate T-cell effector function. Thus, we hypothesized that activation of the CXCR4–CXCL12–STAT3 pathway in CLL cells, leading to IL-10 production, may provide an important mechanism by which CLL cells elaborate their immunoregulatory function. To test this prediction, we first cultured negatively selected CD3+ T cells from healthy controls with purified CLL cells at a 1:10 ratio for 48 h, in the presence or absence of 250 ng/ml of CXCL12. As shown in Figures 3 and 4 CLL cells cultured with CXCL12 induced significantly greater suppression of CD3+ T-cell function, including TNF-α, IFN-γ and IL-2 production, as well as cytotoxicity as measured by CD107a degranulation, in comparison with CD3+ T cells cultured with untreated CLL cells or with CXCL12 alone (Figure S6 in Supplementary Material). In addition, CLL cells cultured with CXCL12 inhibited T cell proliferation in comparison with T cells cultured with untreated CLL cells (Figure S7 in Supplementary Material). Priming CLL cells with CXCR4 blocking antibodies or the addition of blocking antibodies against IL-10 to the coculture of CXCL12-treated CLL and T cells completely reversed T cell dysfunction (Figures 3 and 4). These results suggest that CLL B cells harbor a subpopulation with the ability to regulate T cell effector function through secretion of IL-10, induced by CXCL12–CXCR4 interaction.

**Figure 5:**
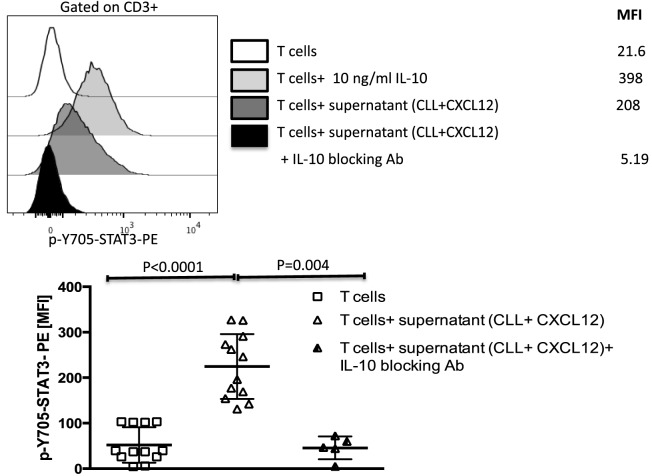
The CXC chemokine ligand 12 (CXCL12)-mediated IL-10 production by chronic lymphocytic leukemia (CLL) cells induces T-cell suppression through phosphorylation of Y705-STAT3 in T cells. IL-10 from CLL cells induces phosphorylation of Y705-STAT3 in T cells. T cells were stimulated with 10 ng/ml of IL-10 or with supernatant from the coculture of CLL+CXCL12 for 20 min, fixed, permeabilized, and stained for p-Y705-STAT3 (*n* = 12).

IL-10 binds to IL-10 receptor on the surface on T cells, inducing phosphorylation of STAT3 on the 705-tyrosine residue (34). We, thus, asked if the STAT3 pathway is also involved in mediating CLL-induced T cell dysfunction. To address this question, we cultured healthy donor T cells for 20 min in supernatants collected from CXCL12-treated CLL cells and tested for activation of the STAT3 pathway. Exogenous IL-10 was used as a positive control. Supernatant collected from the coculture of CLL cells with CXCL12 (Figure 5), but not from CLL cultured alone or exogenous CXCL12 (data not shown), induced phosphorylation of Y705-STAT3 in healthy T cells. The addition of an IL-10 blocking antibody to the coculture fully prevented phosphorylation of Y705-STAT3 in T cells (Figure 5). In agreement with earlier reports (35, 36), these findings indicate that IL-10 secretion by B-CLL cells activates Y705-STAT3, leading to T cell suppression.

### Lenalidomide Can Reverse CLL-Induced T Cell Dysfunction by Inhibiting Both CXCL12-Mediated IL-10 Production by CLL B Cells and Y705-STAT3 Phosphorylation in T Cells *In Vitro*

**Figure 6:**
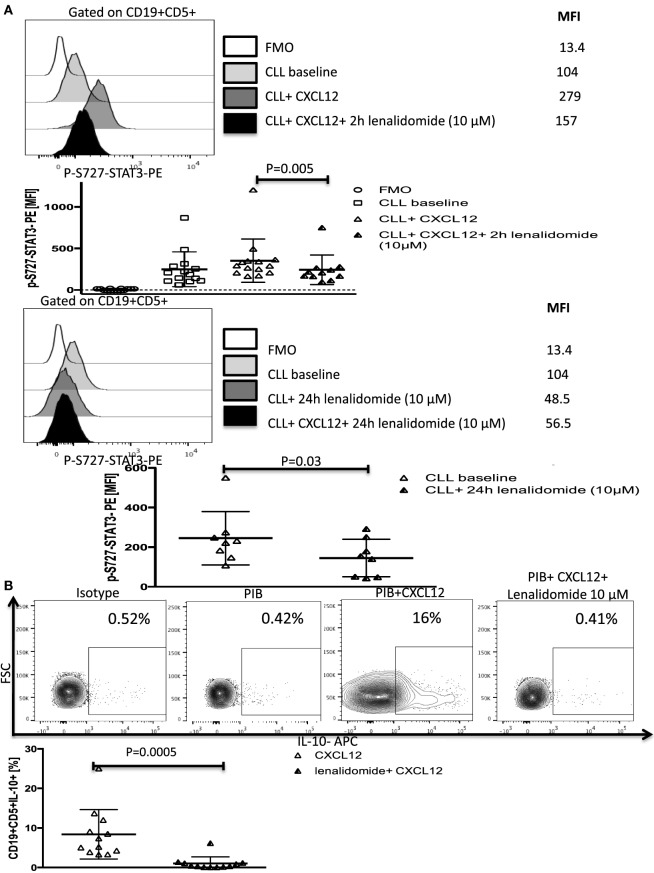
Lenalidomide can reverse chronic lymphocytic leukemia (CLL)-induced T-cell dysfunction by inhibiting CXC chemokine ligand 12 (CXCL12)-mediated IL-10 production by CLL cells. **(A)** Lenalidomide exposure reverses CXCL12-induced S727-STAT3 phosphorylation in CLL cells. CLL cells were incubated with 10 µM lenalidomide for 2 (*n* = 10) or 24 h (*n* = 8) at 37°C and then stimulated with 250 ng/ml CXCL12 for 20 min, fixed, permeabilized, and stained for p-S727-STAT3. **(B)**. Lenalidomide exposure reverses CXCL12-induced IL-10 production by CLL cells. CLL cells were cultured for 2 h with 10 µM lenalidomide. The cells were then stimulated with CXCL12, fixed, and stained according to our IL-10 staining protocol (*n* = 11).

Lenalidomide, a thalidomide derivative, is an immune-modulatory drug with efficacy in patients with CLL (37, 38). Although lenalidomide was shown to improve antitumor immunity (4, 39), its mechanisms of action remain unclear. Recent reports that lenalidomide can inhibit STAT3 phosphorylation (40, 41) and improve T-cell synapse formation and function in CLL (4, 42) led us to investigate its effect on the CXCL12/CXCR4/STAT3/IL-10/T-cells axis. We first investigated whether it can inhibit CXCL12-induced phosphorylation of S727-STAT3. In subsequent experiments, we treated CLL cells with lenalidomide *in vitro* and measured p-S727-STAT3 levels after CXCL12 stimulation. Treatment of CLL cells with the optimal concentration of lenalidomide (10 µM) as measured by a dose titration assay (Figure S8 in Supplementary Material) prevented CXCL12-induced increase in p-S727-STAT3 above baseline as measured by phosflow (Figure 6A; Figure S8 in Supplementary Material) and Western blotting (Figure S9 in Supplementary Material), and resulted in a significant reduction in the IL-10 response by B-CLL cells as well as the baseline constitutive phosphorylation of S727-STAT3 (Figures 6A,B). Lenalidomide did not affect the levels of total STAT3 (Figure S3 in Supplementary Material).

**Figure 7:**
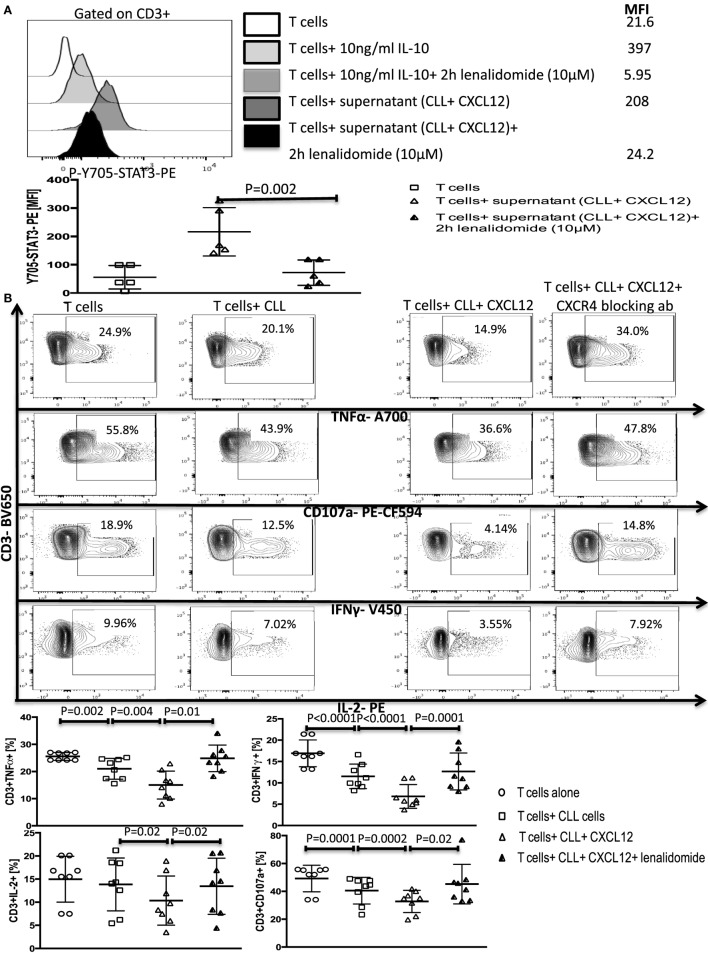
Lenalidomide can reverse chronic lymphocytic leukemia (CLL)-induced T-cell dysfunction by inhibiting IL-10 induced Y705-STAT3 phosphorylation in T cells. **(A)** Lenalidomide exposure prevents IL-10-induced Y705-STAT3 phosphorylation in T cells. T cells were stimulated with 10 ng/ml IL-10 or with the supernatant from CLL+CXC chemokine ligand 12 (CXCL12) cultures in the presence or absence of lenalidomide. Cells were then fixed and stained for p-Y705-STAT3 phosflow (*n* = 5). **(B)** Lenalidomide protects T cells from CLL-induced suppression. T cells were cocultured with CLL cells (1:10 ratio) alone or with 250 ng/ml CXCL12 in the presence or absence of 10 µM lenalidomide for 48 h. T cells were then isolated and stimulated with anti-CD3/CD28 beads for 6 h and stained for IFNγ, TNFα, IL-2, and CD107a, gated on CD3+ cells (*n* = 8).

We next asked if lenalidomide can also suppress IL-10-induced Y705-STAT3 phosphorylation in healthy T cells, thereby inhibiting CLL-induced T-cell dysfunction. Treatment of healthy donor T cells with the drug for 2 h prevented the IL-10-mediated phosphorylation of Y705-STAT3 by exogenous IL-10 or CXCL12–CLL supernatant (Figure 7A). Next, lenalidomide was added (10 µM) to cocultures of healthy donor T cells and CLL cells (1:10 ratio) for 48 h in the presence of 250 ng/ml of CXCL12. Lenalidomide prevented CLL-induced T cell dysfunction as measured by the production of IFN-γ, TNF-α, and IL-2 and CD107 degranulation (Figure 7B). Lenalidomide alone had no effect on healthy T cells function (Figure S10 in Supplementary Material). These findings indicate that lenalidomide can reverse CLL-induced immunosuppression through abrogation of the CXCL12–CXCR4–IL-10–STAT3 response in CLL cells and also prevention of T-cell suppression through inhibition of the IL-10-induced phosphorylation of Y705-STAT3.

### Treatment with Lenalidomide Blocks CXCL12-Induced IL-10 Production by B-CLL Cells and Reverses the Associated T-Cell Dysfunction *In Vivo*

**Figure 8:**
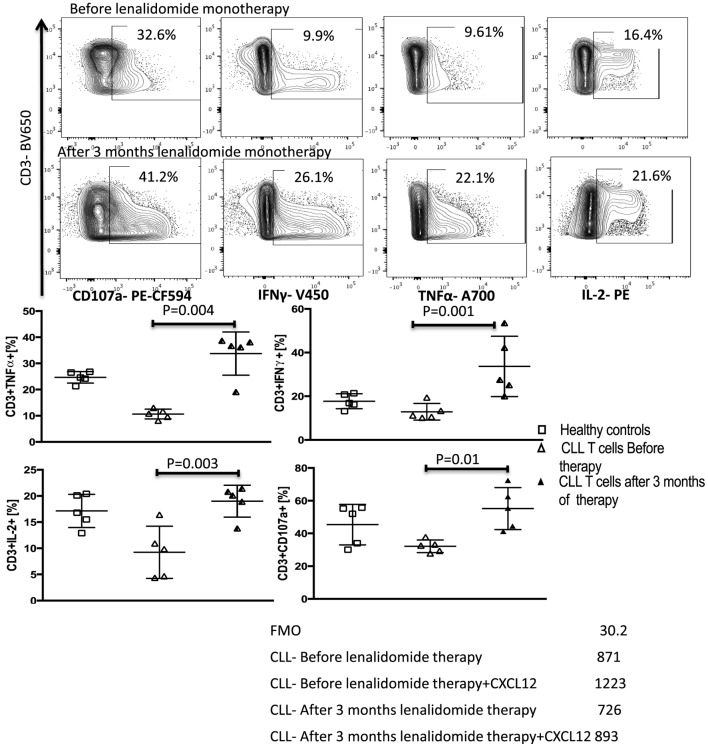
Lenalidomide reverses chronic lymphocytic leukemia (CLL)-induced T-cell dysfunction *in vivo*. Peripheral blood mononuclear cells (PBMCs) were collected from five patients treated with lenalidomide monotherapy (clinical trial 2006-0715, NCT00535873) before and after 3 months of treatment. T-cell function improved after lenalidomide monotherapy. T cells were stimulated with anti-CD3/CD28 beads for 6 h and stained for IFN-γ, TNF-α, IL-2, and CD107a. FACS plots are gated on live CD3+ cells.

**Figure 9:**
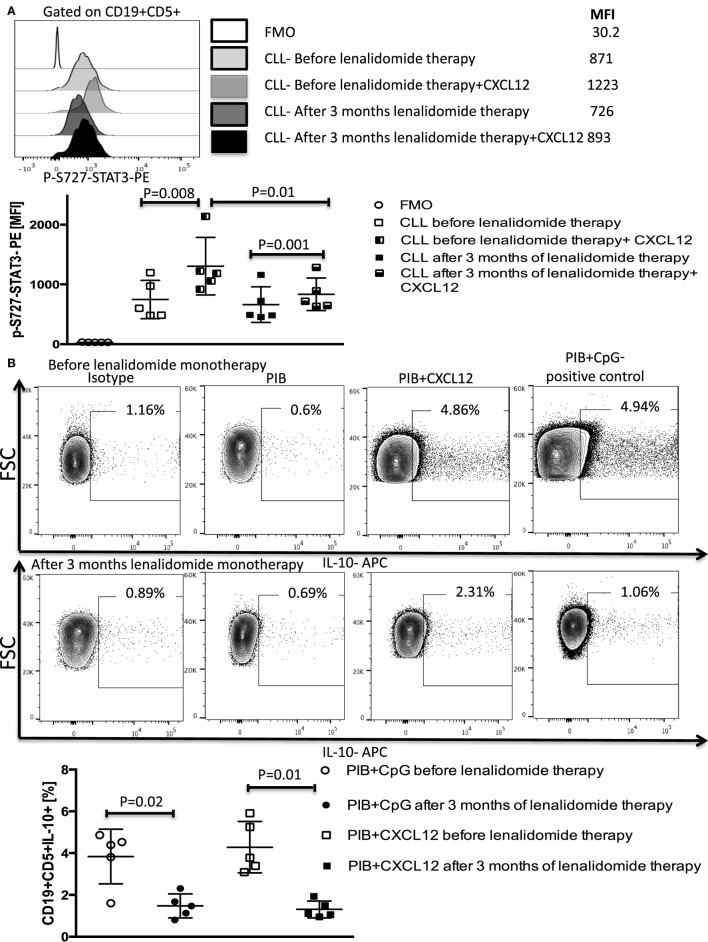
Lenalidomide blocks CXC chemokine ligand 12 (CXCL12)-induced S727-STAT3 phosphorylation and IL-10 production by chronic lymphocytic leukemia (CLL) cells *in vivo*. PBMCs were collected from five patients treated with lenalidomide monotherapy (clinical trial 2006-0715, NCT00535873) before and after 3 months of treatment. **(A)** Lenalidomide therapy reduced CXCL12-induced p-S727-STAT3 in CLL cells. CLL cells were cultured in the presence or absence of 250 ng/ml of CXCL12 for 20 min. The cells were then fixed, permeabilized, and stained for p-S727-STAT3. **(B)** Lenalidomide therapy reduces CXCL12-induced IL-10 production by CLL cells. CLL cells were stimulated with 250 ng/ml CXC12 and cultured for 8–10 h, followed by addition of phorbol myristate acetate (PMA), ionomycin, BFA (PIB), then incubated for 6 h before performing intracellular staining.

We next sought to substantiate the mechanism shown *in vitro* that lenalidomide reverses T-cell dysfunction by preventing IL-10 production by CLL cells and IL-10-induced phosphorylation of Y705-STAT3 in T cells, using peripheral blood samples collected and cryopreserved from patients treated with lenalidomide monotherapy. Details of this clinical trial (Clinical trial 2006-0715, NCT00535873) were reported in a previous publication (37). The patient characteristics are summarized in Table 2. Briefly, patients were treated with lenalidomide at a median daily dose of 5 mg (range 2.5–10 mg) for 28 days per cycle for 3–6 cycles (90 days). PBMCs were collected and cryopreserved before drug treatment and during the first 3 months of therapy. Analysis of S727-STAT3 phosphorylation in CLL cells, IL-10 production by CLL cells in response to CXCL12 stimulation, and T-cell function before and during lenalidomide treatment showed that the drug improved T-cell dysfunction (Figure 8). Moreover, lenalidomide treatment prevented a CXCL12-induced increase in S727-STAT3 phosphorylation in CLL cells over baseline (Figure 9A) and, thus, IL-10 production by CLL cells (Figure 9B). We concluded that the antileukemic activity of lenalidomide in CLL operates is at least partly through modulation of the STAT3 pathway and inhibition of IL-10 production by CLL-B cells.

**Table 2:** Patient characteristics for chronic lymphocytic leukemia patients treated with lenalidomide.

Median age (range)	72 years (66–78)
**Gender:**	
Female	1 (20%)
Male	4 (80%)
**Rai stage:**	
0	0
1	1 (20%)
2	0
3	1 (20%)
4	3 (60%)
**Zap70:**	
Zap70−	2 (40%)
Zap70+	0
Unknown	3 (60%)
**IgV_H_ mutation status:**	
IgH_H_−	1 (20%)
IgV_H_+	3 (60%)
Unknown	1 (20%)
**Cytogenetics:**	
13q	3 (60%)
t12	2 (40%)
TP53	1 (20%)
No mutations	1 (20%)
Unknown	0
**Secondary malignancies:**	
Yes	0
No	5 (100%)
Previous therapy	2 (40%)
No previous therapy	3 (60%)
Response to lenalidomide	Partial remission (PR) 5 (100%)

## Discussion

Immunosuppression is a major complicating factor in the disease course of CLL, leading to severe infections, increased risk of secondary malignancies, and death (1, 43). The mechanisms of CLL-induced immune dysfunction remain unclear but likely involve complex interactions among leukemic cells, the tumor microenvironment and immune effectors (6–10). Recently, DiLillo et al. (21) reported the existence of a subset of IL-10-producing CLL cells, similar to regulatory B10 cells. While Bregs can inhibit T_H1_ function and monocyte activation (11, 13, 14), the impact of these cells on the immune function in CLL remains unclear. Here, we show that the CXCL12–CXCR4–STAT3 axis regulates IL-10 production by CLL cells and their ability to suppress T-cell effector function. This mechanism was suggested by the heterogeneous functions of CXCL12, which is constitutively secreted by CLL stroma cells. CXCL12 binds CXCR4 on CLL cells, is a dominant factor in the homing of CLL cells to bone marrow, and activates different intracellular signaling cascades associated with chemotaxis and leukemia cell survival, including STAT3 (6, 9, 23, 44). This is in accordance with recent reports associating high CXCL12 levels in the sera of CLL patients with poor prognosis (45). STAT3 can be transcriptionally activated by phosphorylation of its tyrosine 705 or serine 727 residues. Like tyrosine phosphorylated STAT3 in B cells (12–15), phosphorylated S727-STAT3 in CLL cells can translocate to the nucleus, bind to DNA, and activate genes involved in regulating survival and proliferation (31), as well as induce expression of IL-10 gene (46). Although CXCL12 has been previously linked to T-cell immune suppression in cancer (47) and even to IL-10 production in T cells (48, 49) and tissue-infiltrating B cells (50), this is the first report to investigate an immunomodulatory function of CXCL12 in CLL and propose an underlying mechanism for IL-10 production by CXCL12. Thus, our demonstration that CXCL12–CXCR4 interaction induces phosphorylation of S727-STAT3, leading to IL-10 secretion by B-CLL cells and then to IL-10-induced Y705-STAT3-phosphorylation in T cells, resulting in their suppression, represents a novel route to immunosuppression associated with CLL.

Exactly, how these insights could be exploited to improve therapy for CLL is unclear, but inhibition of the binding of CXCL12 and CXCR4 provides an attractive starting point. The CXCR4 antagonist, plerixafor, is currently in clinical testing in CLL (NCT00694590 and NCT01373229). Further trials in CLL are warranted to assess the value of this agent in correcting immune dysfunction and slowing disease progression.

Lenalidomide is a known immunomodulator with efficacy against CLL (37, 39) and other hematologic malignancies, including multiple myeloma (51) and myelodysplastic syndromes (52). Yet, the mechanism of action of lenalidomide as it relates to immune modulation remains elusive. Based on findings in preclinical models and clinical trials, lenalidomide is thought to enhance the proliferation and functionality of T lymphocytes, amplify costimulatory signaling pathways that activate effector responses and improve the strength of synapse formation between T cells and CLL cells (4, 39, 53). Lenalidomide was also recently reported to inhibit IL-6-mediated STAT3 phosphorylation in NK cells (41), decrease CXCL12 expression by CLL stroma cells, and impair CLL response to CXCL12 (54, 55). In addition, it was implicated in the recovery of T-cell proliferation by reducing IL-10 production in CLL macrophages and promoting IL-2 secretion by T-cells (55). Lenalidomide, has also been reported to increase IL-10 production in CLL/nurse like cell cocultures (54), underscoring the complexicity of the CLL microenvironment and the multiple mechanisms involved in immune suppression in this disease.

The concentrations required for CXCL12-induced p-S727-STAT3 inhibition (Between 1 and 10 µM, Figure S8 in Supplementary Material) are clinically achievable, as demonstrated by pharmacokinetics studies (56). This concentration was previously reported to block STAT3 phosphorylation in NK cells and inhibit CXCL12 chemotaxis by CLL cells (41, 54). The mechanistic data we present further elucidate the immunomodulatory mechanism of action of lenalidomide to include abrogation of the CXCL12–CXCR4-mediated IL-10 response by CLL cells and prevention of T-cell suppression due to IL-10-induced phosphorylation of Y705-STAT3.

We, therefore, conclude that identification of CXCL12–CXCR4–STAT3 axis as a pivotal immune-modulatory axis in CLL could provide needed impetus for the design of new therapeutic strategies in CLL or inform different approaches to the use of available immunomodulatory agents, such as lenalidomide in this disease.

## Author Contributions

HS performed experiments, designed, interpreted, analyzed and wrote the manuscript. MHS designed, performed experiments and commented on the manuscript. DH, PR, EG, CS and ZL performed experiments and commented on the manuscript. PAT, AF, MK and WW provided and analyzed clinical data and commented on the manuscript. ZE, LNK, KK, LL, MM, NI, EL, AA, RB, JB, MD and EJS provided advice on experiments and commented on the manuscript. KR designed and directed the study and wrote the manuscript.

## Conflict of Interest Statement

The authors of this manuscript certify that they have no affiliations with or involvement in any organization or entity with any financial interest (such as honoraria; educational grants; participation in speakers’ bureaus; membership, employment, consultancies, stock ownership, or other equity interest; and expert testimony or patent-licensing arrangements), or non-financial interest (such as personal or professional relationships, affiliations, knowledge or beliefs) in the subject matter or materials discussed in this manuscript.
