# Supplementary material for: The CXCR4–STAT3–IL-10 Pathway Controls the Immunoregulatory Function of Chronic Lymphocytic Leukemia and Is Modulated by Lenalidomide
Source: Front Immunol. 2018 Jan 15;8:1773. doi: 10.3389/fimmu.2017.01773 (PMC5775272; doi:10.3389/fimmu.2017.01773)
Supplement: Supplementary file 1 [file Presentation_1.PPTX]

## Slide 1
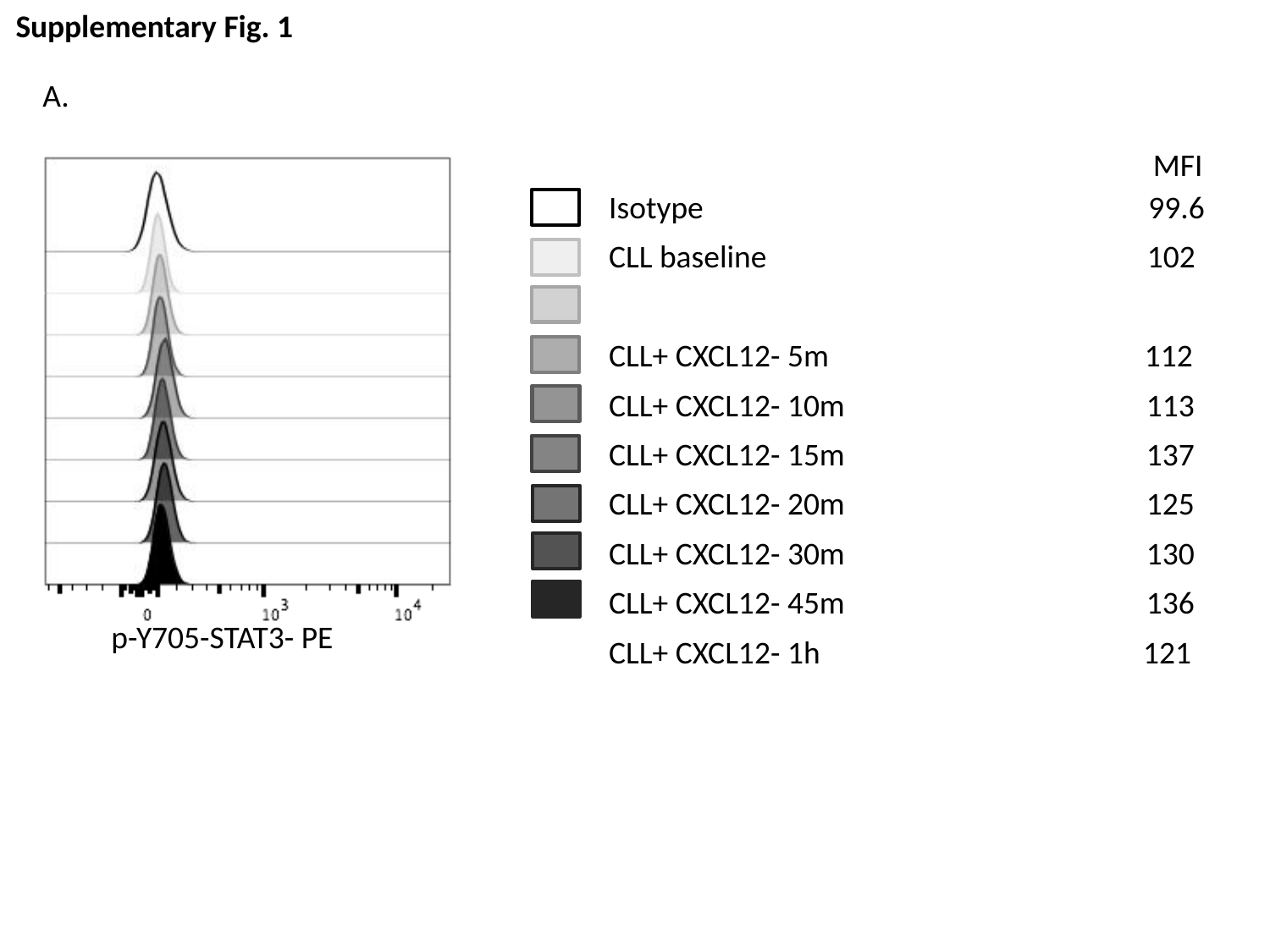

Supplementary Fig. 1
A.
MFI
Isotype 99.6
CLL baseline 102
CLL+ CXCL12- 5m 112
CLL+ CXCL12- 10m 113
CLL+ CXCL12- 15m 137
CLL+ CXCL12- 20m 125
CLL+ CXCL12- 30m 130
CLL+ CXCL12- 45m 136
CLL+ CXCL12- 1h 121
p-Y705-STAT3- PE

## Slide 2
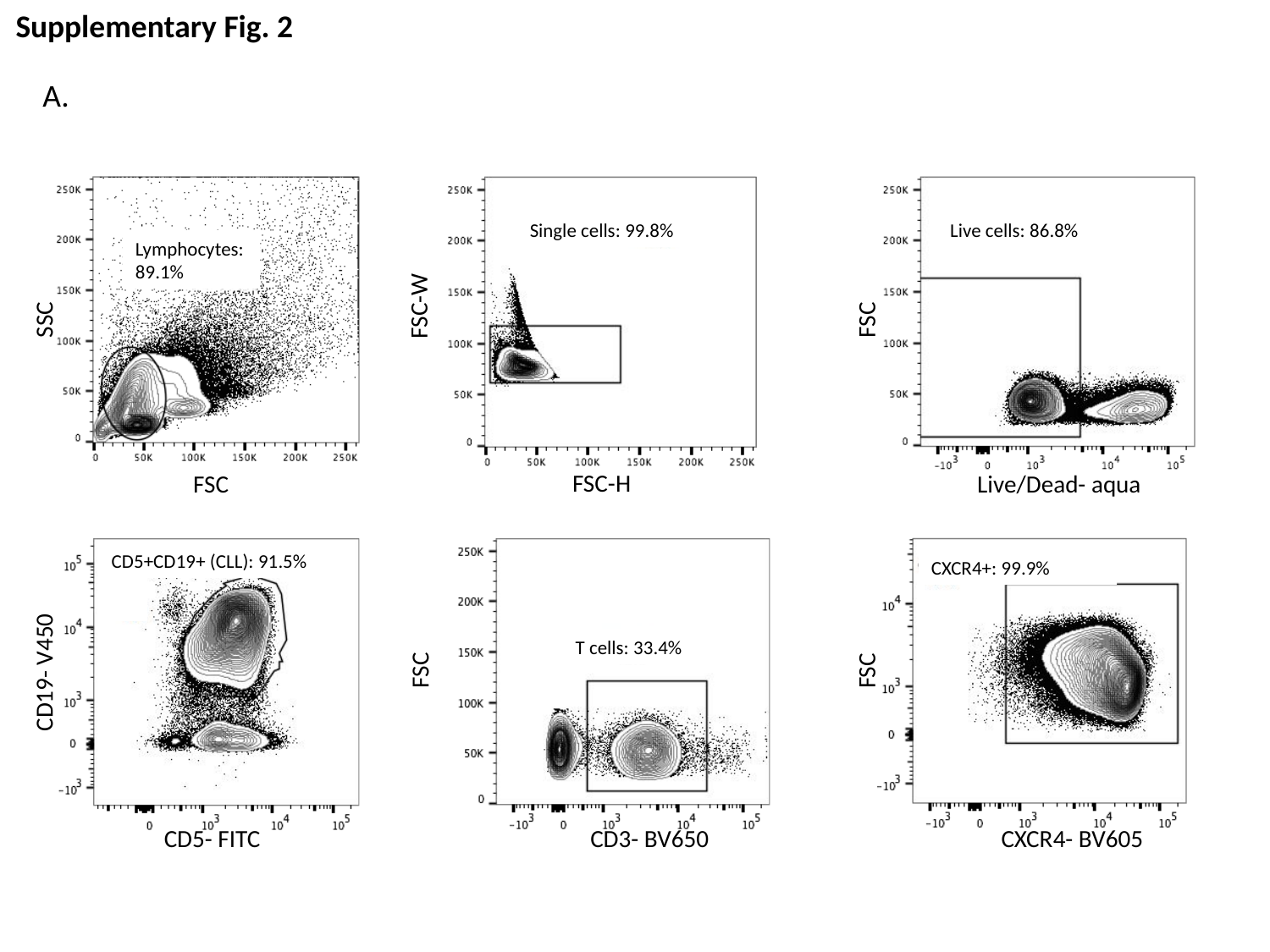

Supplementary Fig. 2
A.
Single cells: 99.8%
Live cells: 86.8%
Lymphocytes:89.1%
SSC
FSC
FSC-W
FSC-H
FSC
Live/Dead- aqua
CD5+CD19+ (CLL): 91.5%
CXCR4+: 99.9%
FSC
FSC
T cells: 33.4%
CD19- V450
CD5- FITC
CD3- BV650
CXCR4- BV605

## Slide 3
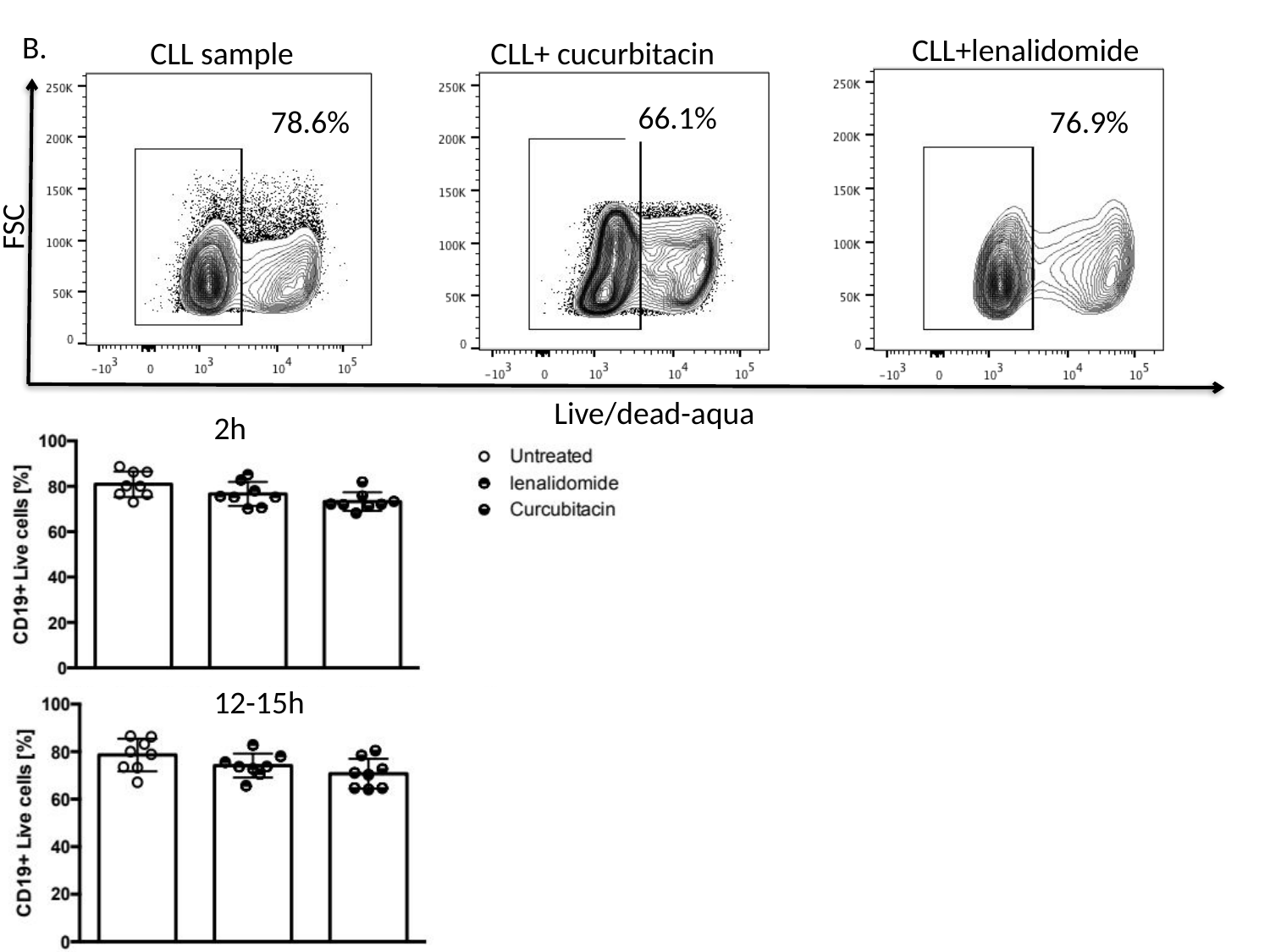

B.
CLL+lenalidomide
CLL sample
CLL+ cucurbitacin
66.1%
78.6%
76.9%
FSC
Live/dead-aqua
2h
12-15h

## Slide 4
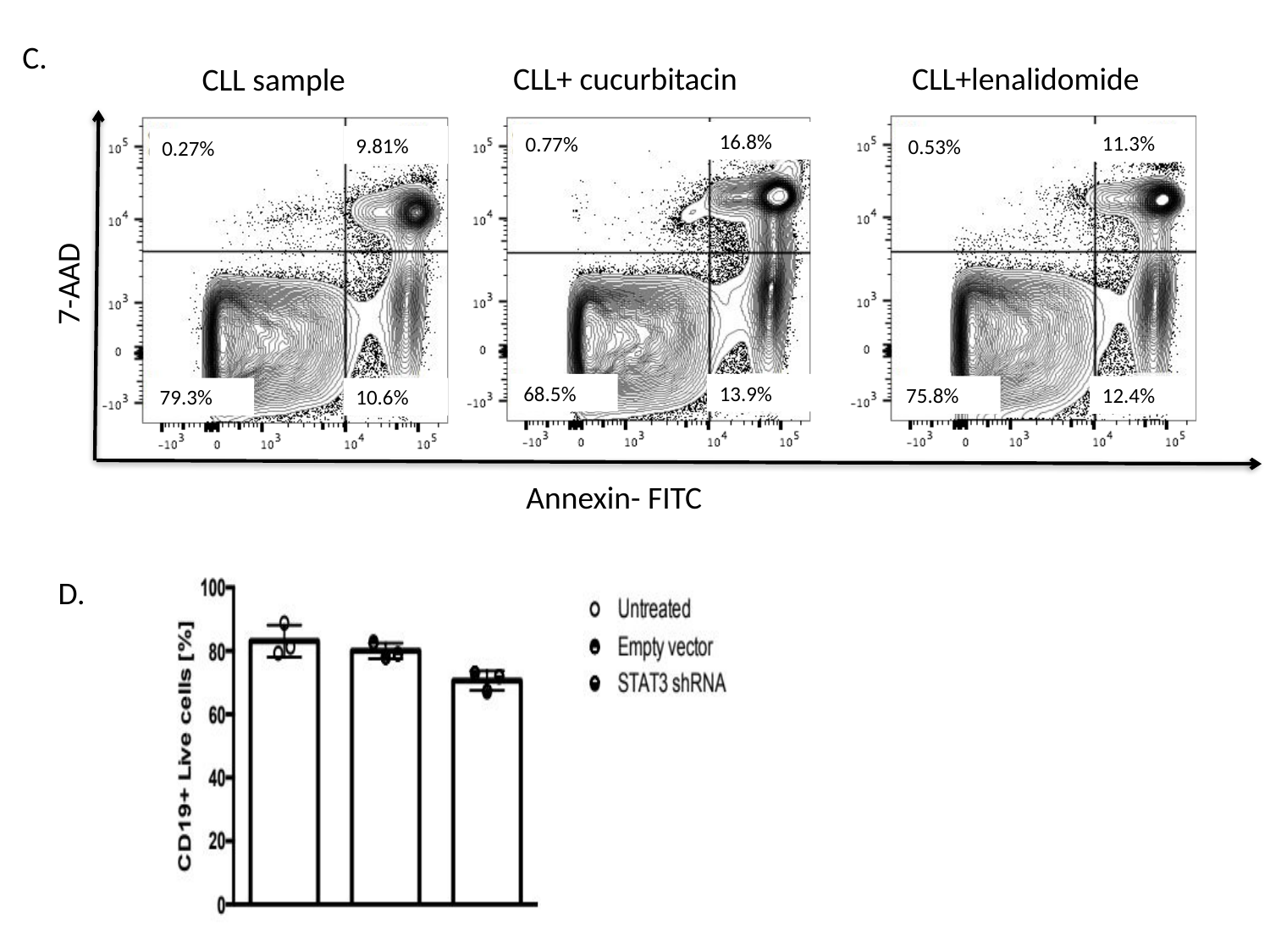

C.
CLL+ cucurbitacin
CLL+lenalidomide
CLL sample
16.8%
11.3%
0.77%
9.81%
0.53%
0.27%
7-AAD
68.5%
13.9%
75.8%
12.4%
79.3%
10.6%
Annexin- FITC
D.

## Slide 5
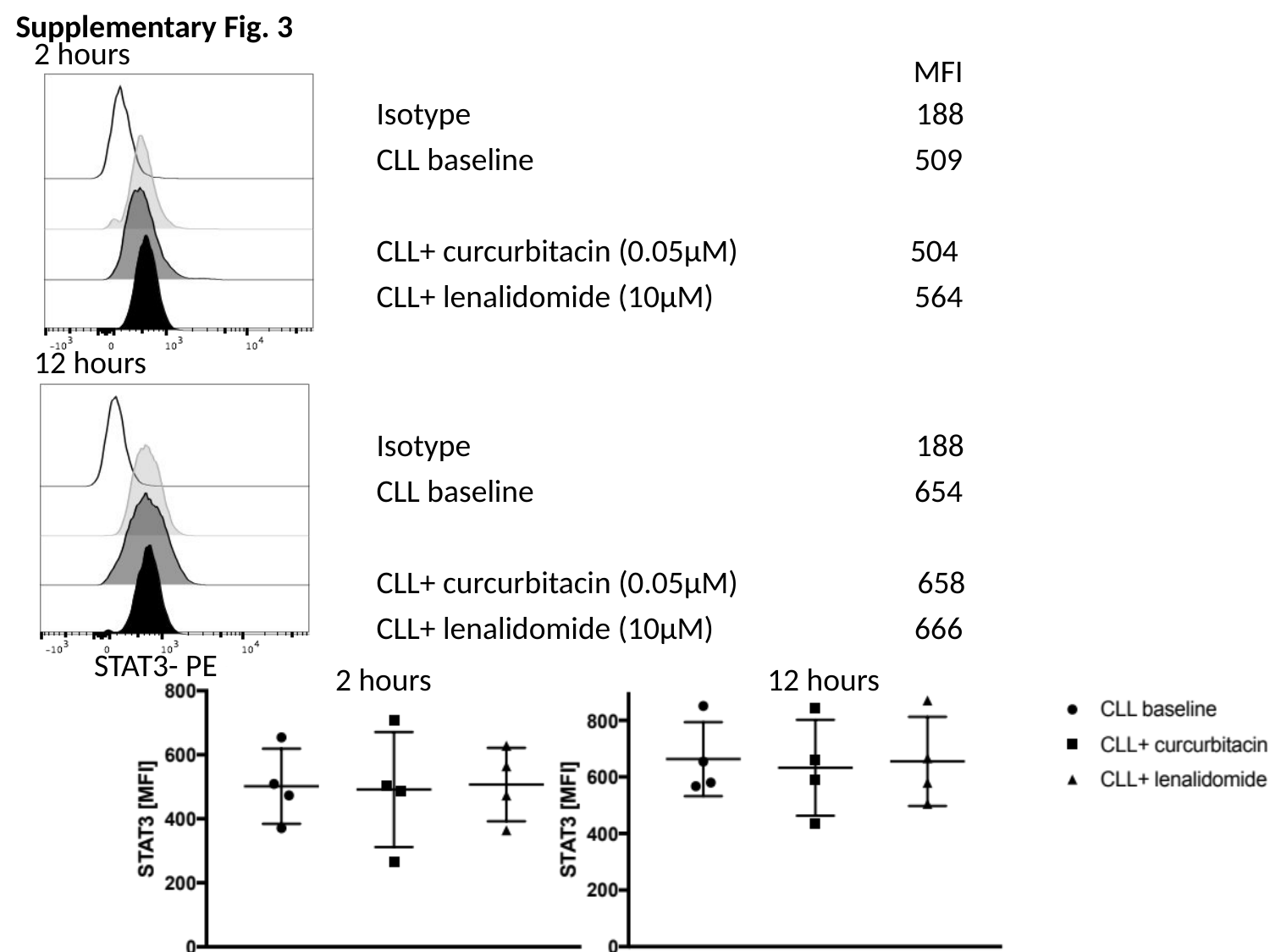

Supplementary Fig. 3
2 hours
MFI
Isotype 188
CLL baseline 509
CLL+ curcurbitacin (0.05μM) 504
CLL+ lenalidomide (10μM) 564
12 hours
Isotype 188
CLL baseline 654
CLL+ curcurbitacin (0.05μM) 658
CLL+ lenalidomide (10μM) 666
STAT3- PE
2 hours
12 hours

## Slide 6
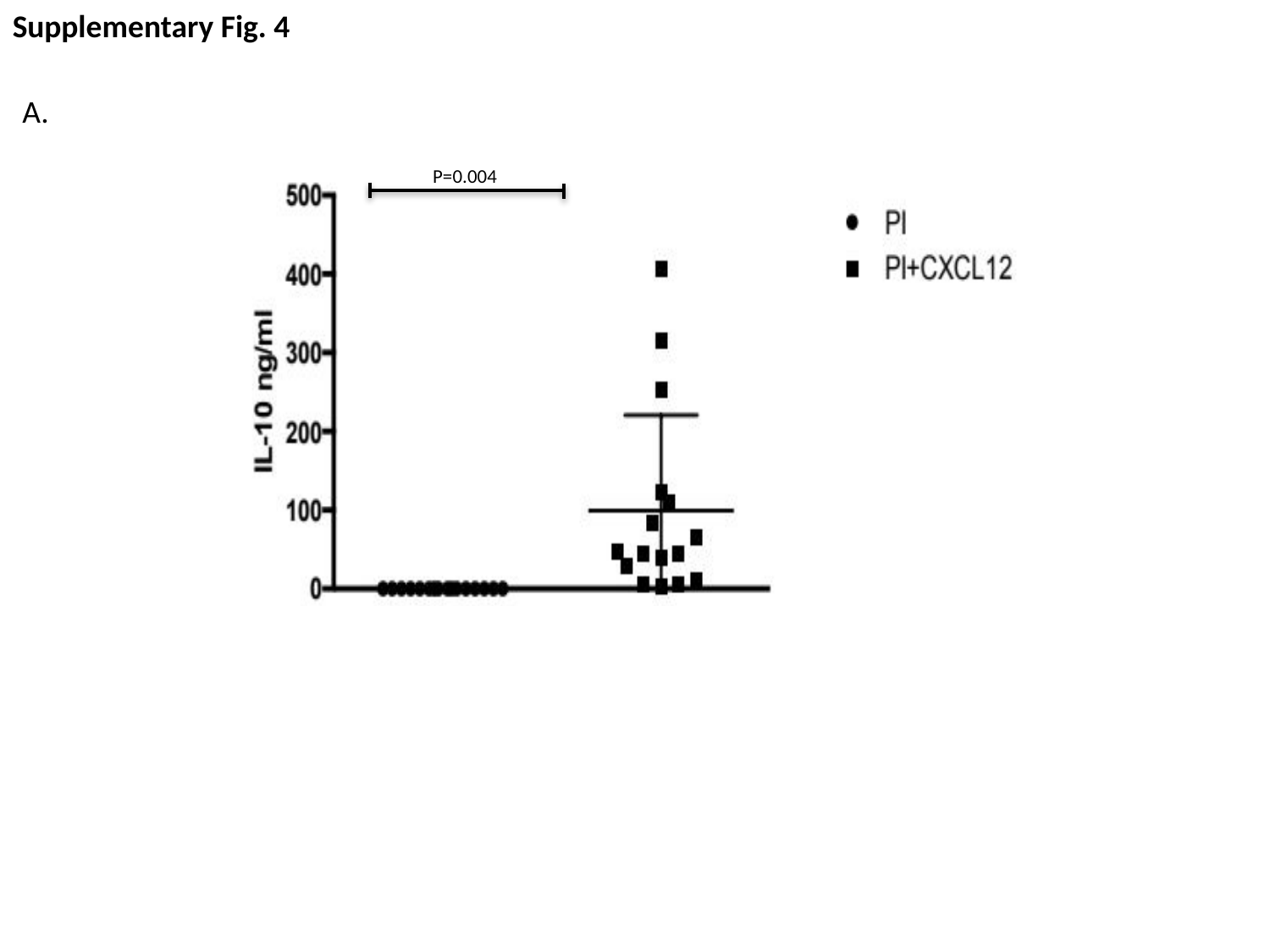

Supplementary Fig. 4
A.
P=0.004

## Slide 7
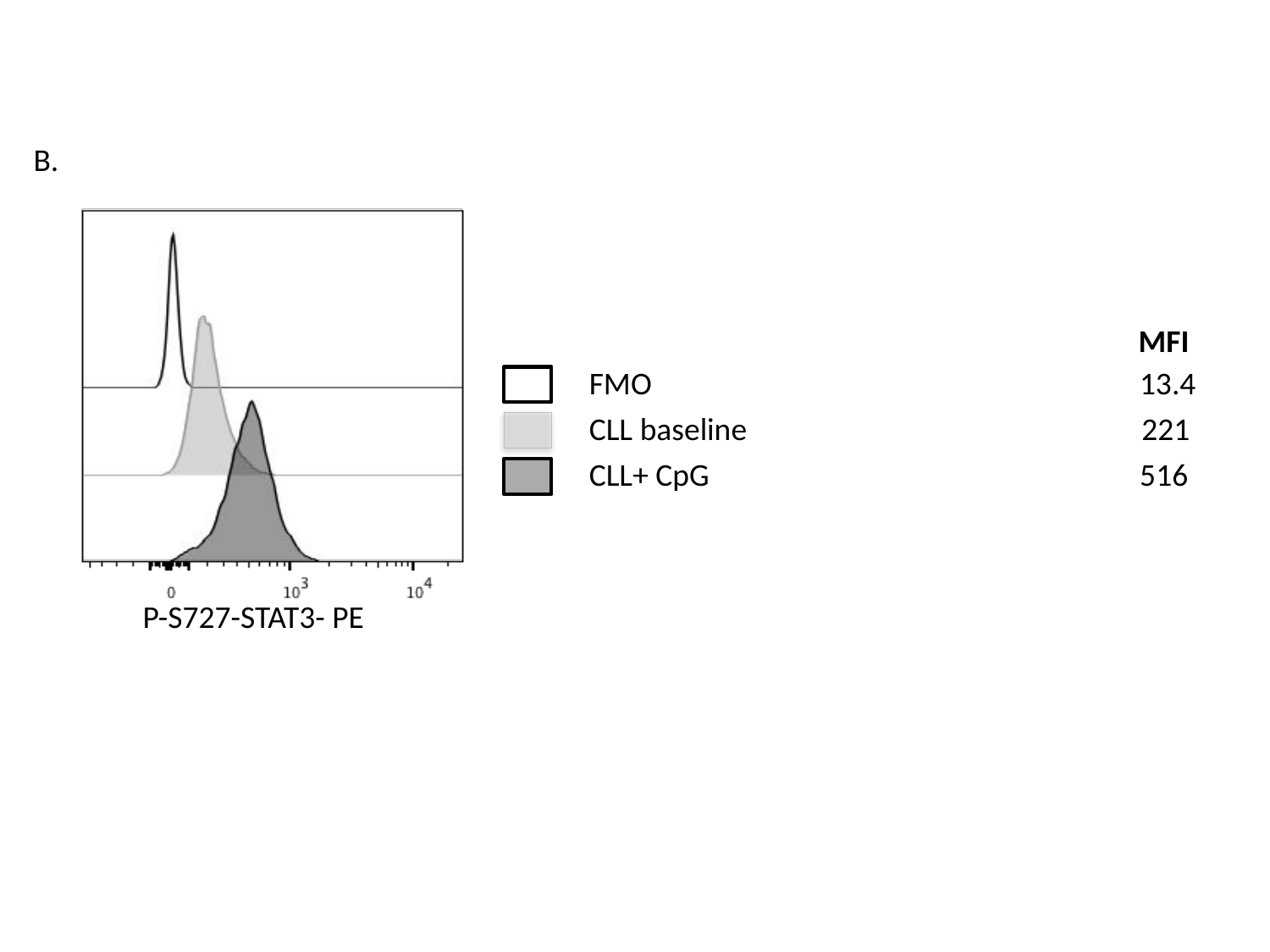

B.
MFI
FMO 13.4
CLL baseline 221
CLL+ CpG 516
P-S727-STAT3- PE

## Slide 8
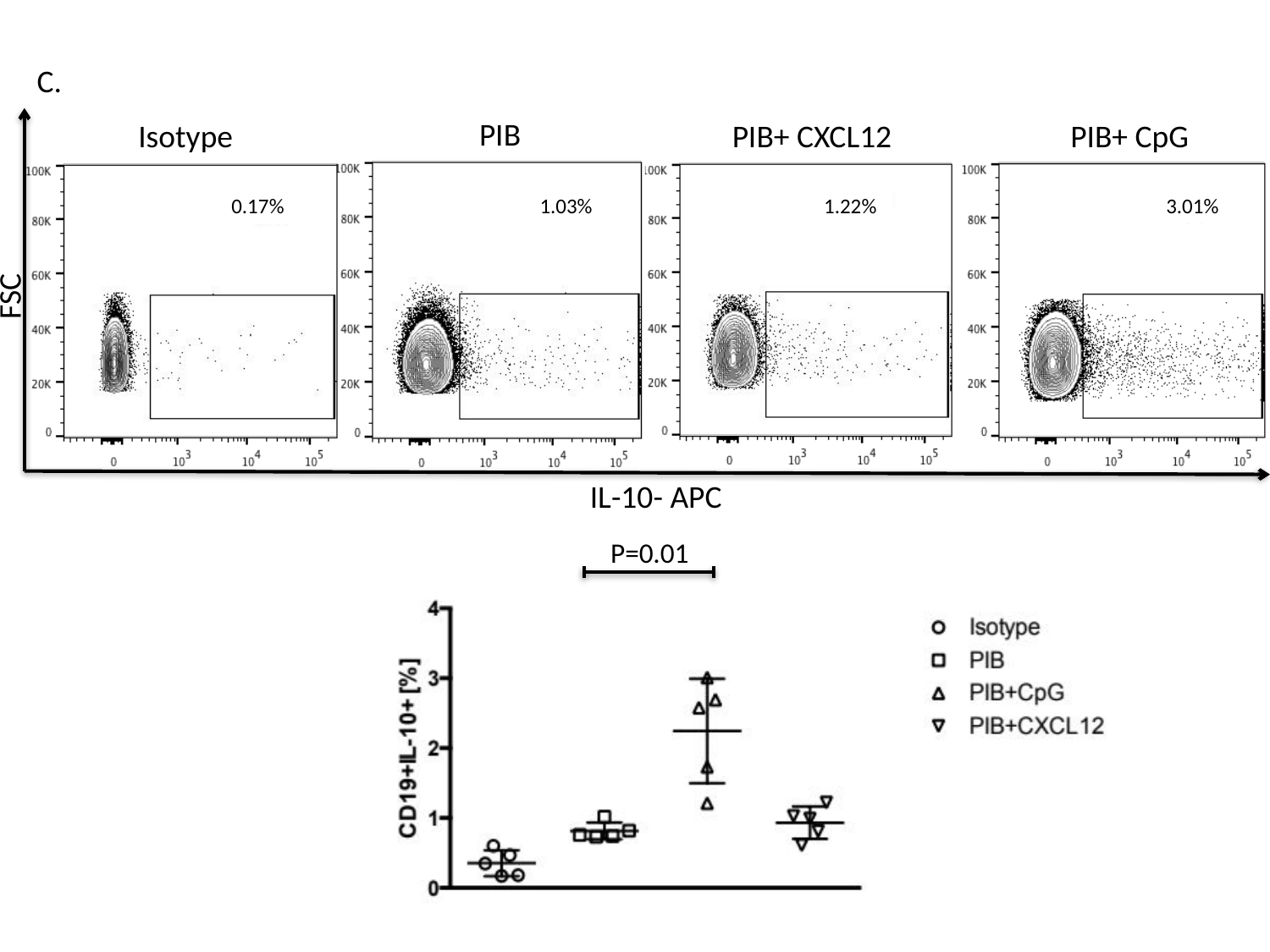

C.
PIB
Isotype
PIB+ CXCL12
PIB+ CpG
1.03%
3.01%
0.17%
1.22%
FSC
IL-10- APC
P=0.01

## Slide 9
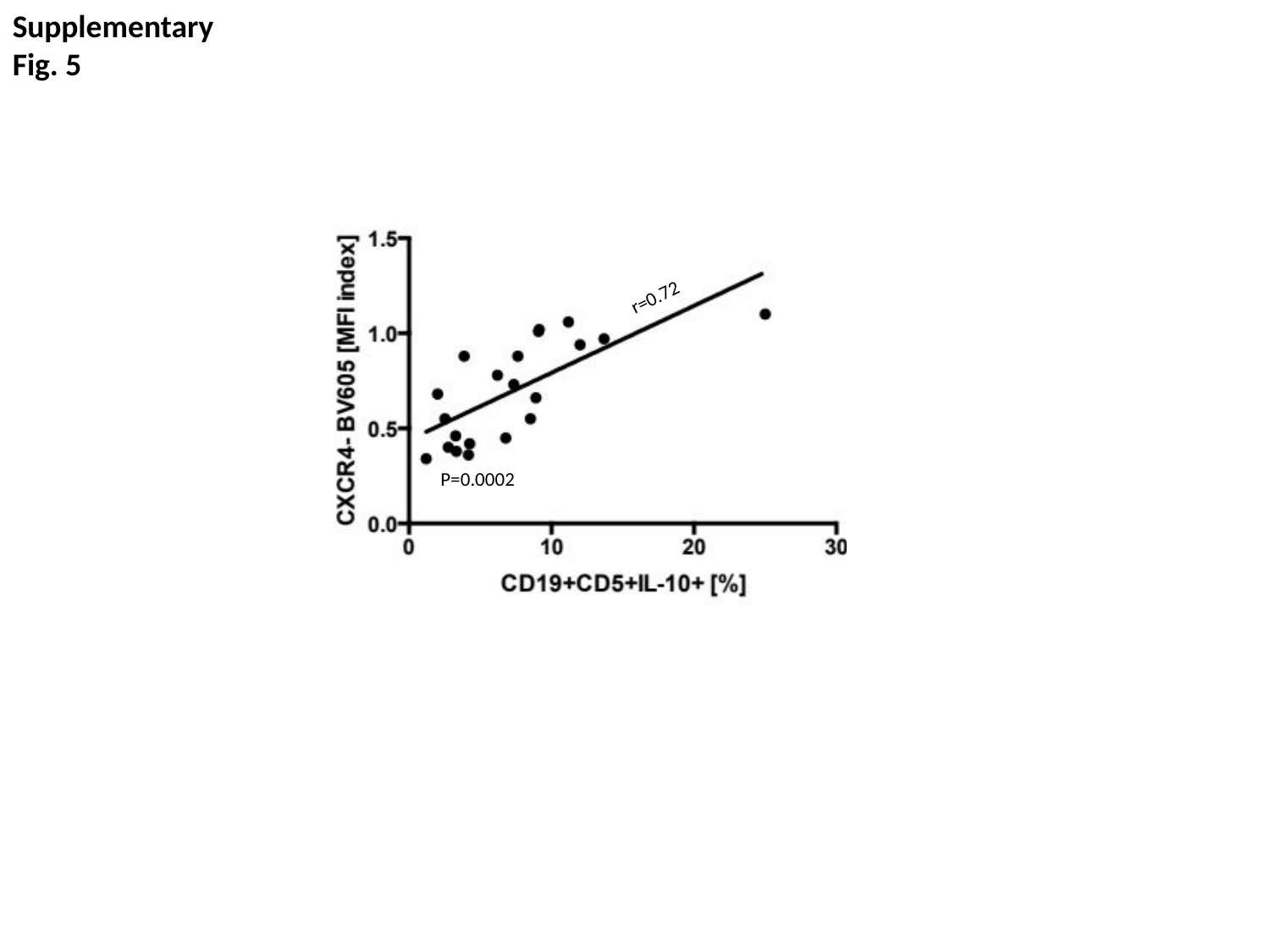

Supplementary
Fig. 5
r=0.72
P=0.0002

## Slide 10
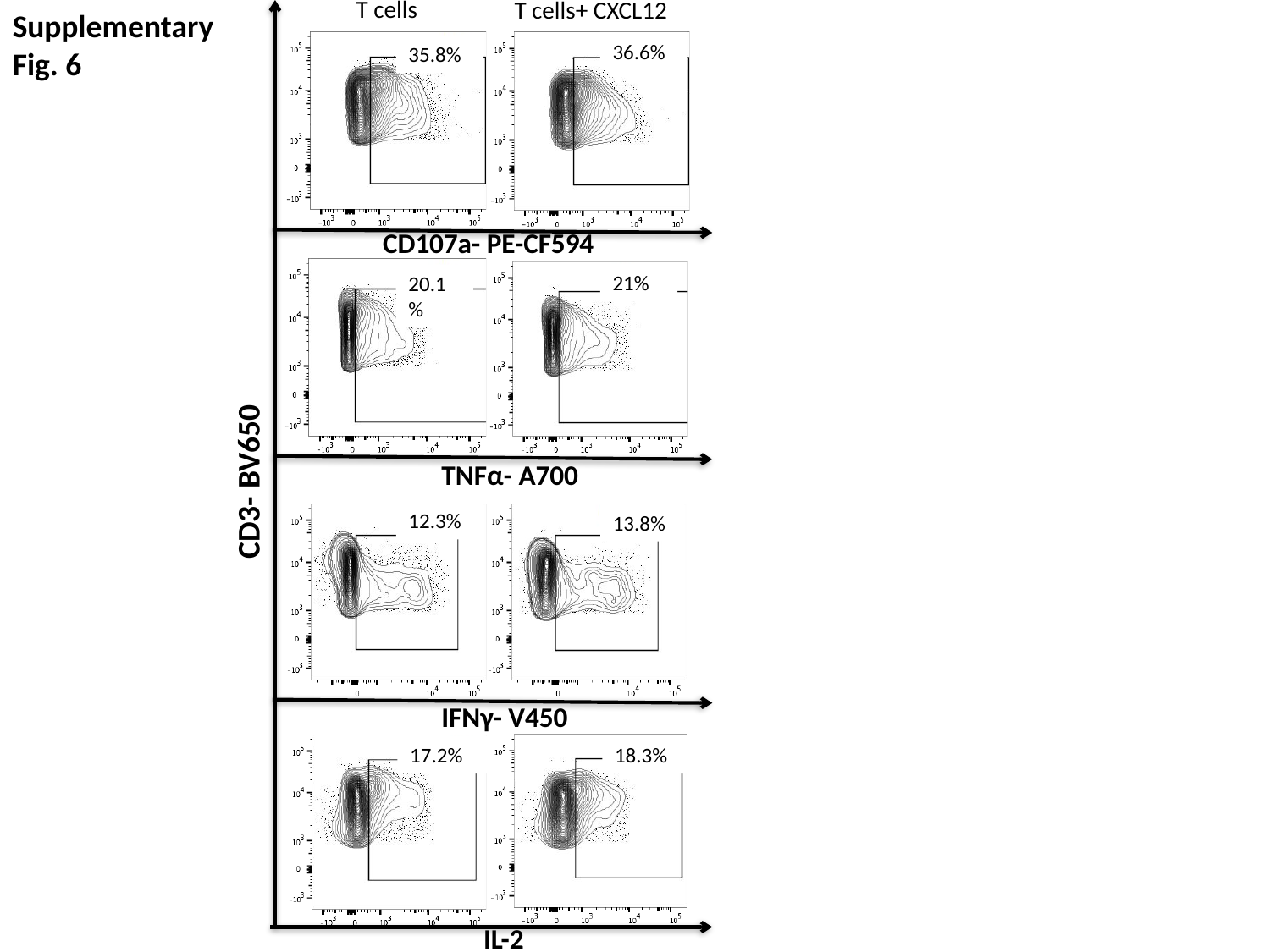

Supplementary
Fig. 6
T cells
T cells+ CXCL12
36.6%
35.8%
CD107a- PE-CF594
21%
20.1%
CD3- BV650
TNFα- A700
12.3%
13.8%
IFNγ- V450
17.2%
18.3%
IL-2

## Slide 11
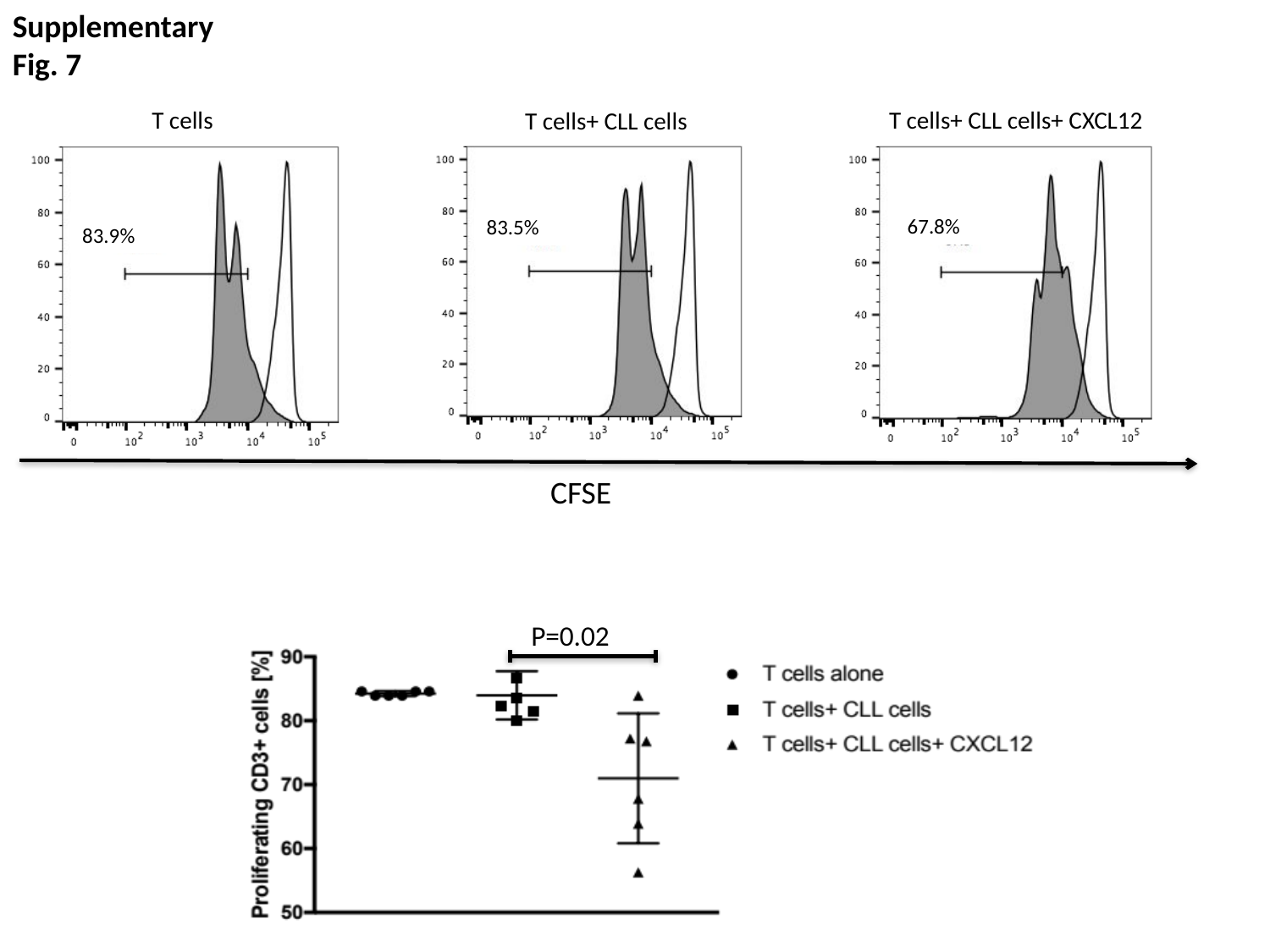

Supplementary
Fig. 7
T cells
T cells+ CLL cells+ CXCL12
T cells+ CLL cells
67.8%
83.5%
83.9%
CFSE
P=0.02

## Slide 12
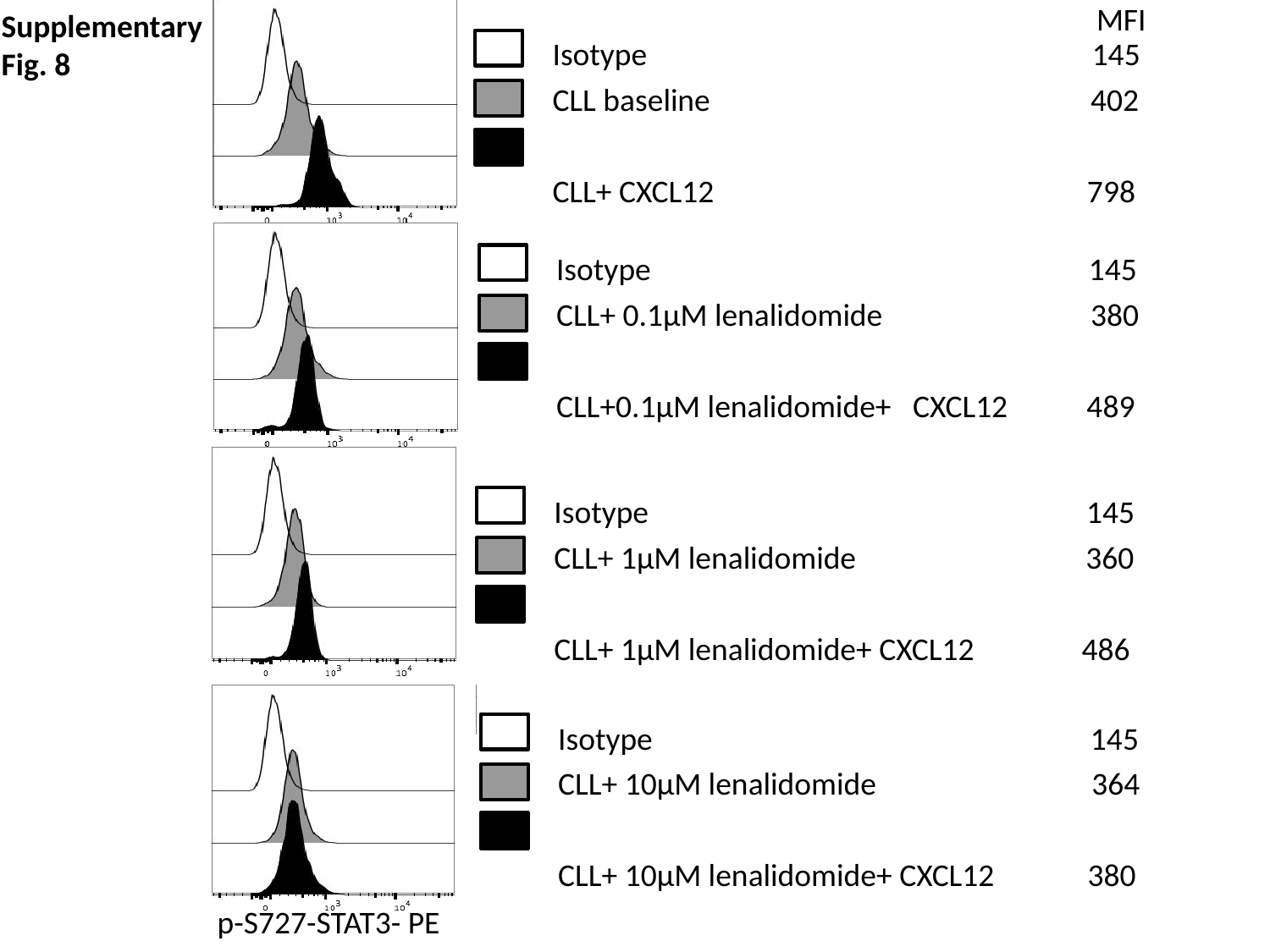

Supplementary
Fig. 8
MFI
Isotype 145
CLL baseline 402
CLL+ CXCL12 798
Isotype 145
CLL+ 0.1μM lenalidomide 380
CLL+0.1μM lenalidomide+ CXCL12 489
Isotype 145
CLL+ 1μM lenalidomide 360
CLL+ 1μM lenalidomide+ CXCL12 486
Isotype 145
CLL+ 10μM lenalidomide 364
CLL+ 10μM lenalidomide+ CXCL12 380
p-S727-STAT3- PE

## Slide 13
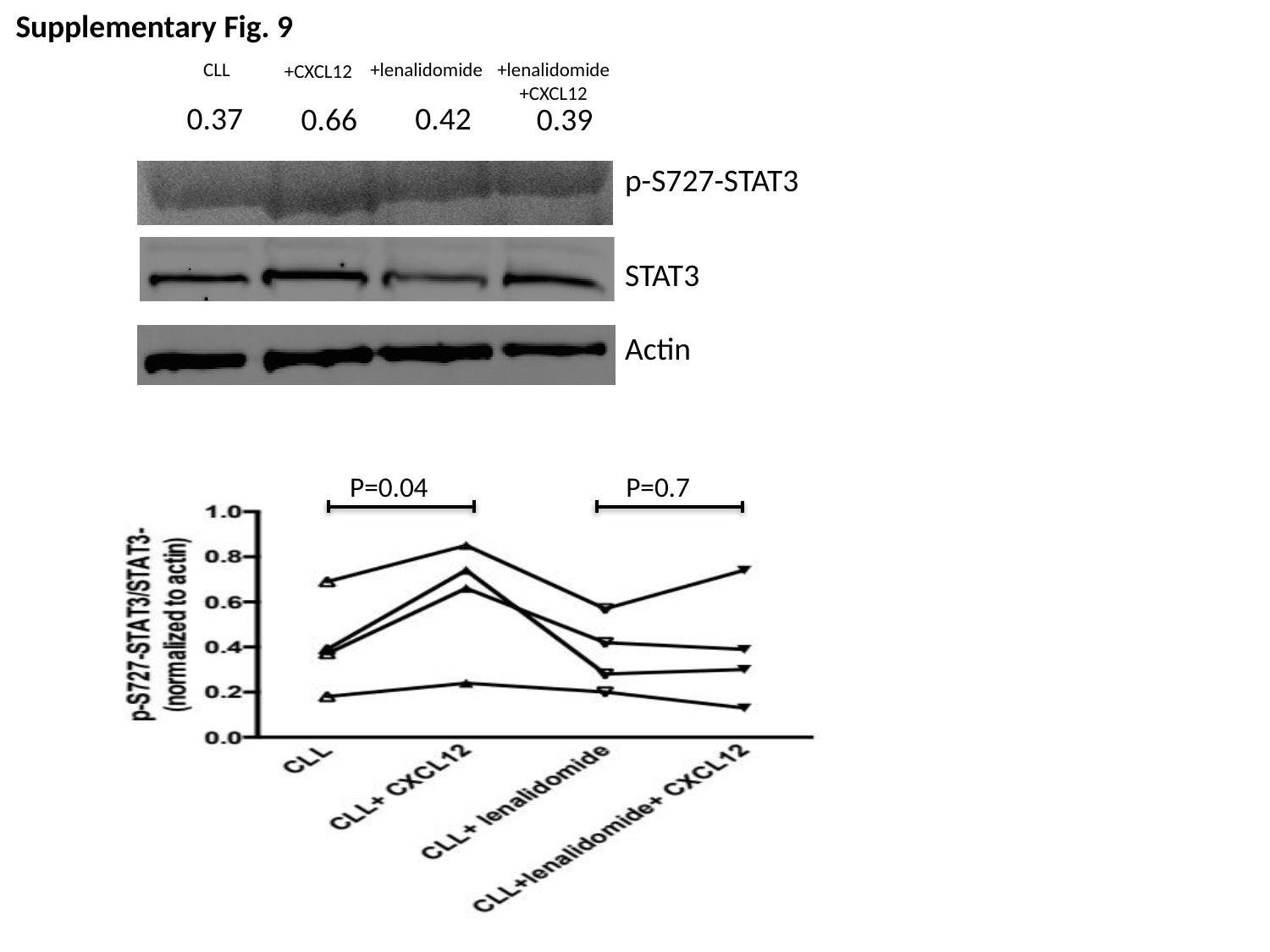

Supplementary Fig. 9
CLL
+lenalidomide
+lenalidomide
+CXCL12
+CXCL12
0.42
0.37
0.66
0.39
p-S727-STAT3
STAT3
Actin
P=0.04
P=0.7

## Slide 14
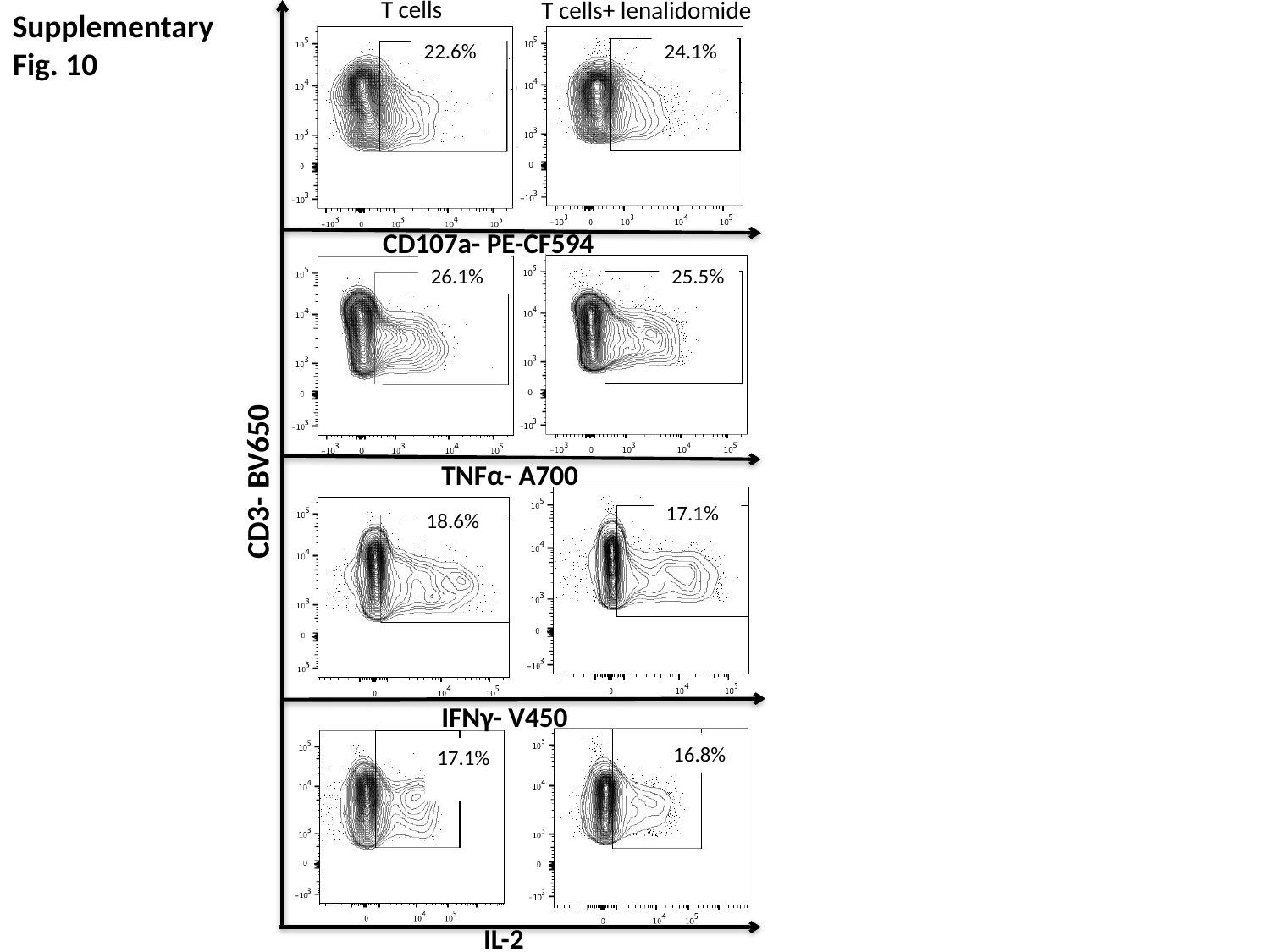

Supplementary
Fig. 10
T cells
T cells+ lenalidomide
24.1%
22.6%
CD107a- PE-CF594
26.1%
25.5%
CD3- BV650
TNFα- A700
17.1%
18.6%
IFNγ- V450
16.8%
17.1%
IL-2
