# Supplementary material for: The CXCR4–STAT3–IL-10 Pathway Controls the Immunoregulatory Function of Chronic Lymphocytic Leukemia and Is Modulated by Lenalidomide
Source: Front Immunol. 2018 Jan 15;8:1773. doi: 10.3389/fimmu.2017.01773 (PMC5775272; doi:10.3389/fimmu.2017.01773)
Supplement: Supplementary file 2 [file Data_Sheet_1.DOCX]

**Supplementary figures**

**Supplementary Fig 1. CXCL12 does not induce phosphorylation of the tyrosine 705 residue of STAT3 in CLL cells.** CLL cells were stimulated with 250 ng/ml CXCL12 and pY705-STAT3 was measured by Phosflow as described in materials and methods.

**Supplementary Fig 2**. **CLL viability following in vitro culture with lenalidomide or cucurbitacin, or following transfection with STAT3-shRNA. (A)** Representative FACS plots of the gating strategy for flow cytometry. **(B-C)** The viability after 2 hours or 12-15 hours culture with 10 μM lenalidomide or 0.05 μM cucurbitacin was assessed with live/dead-aqua staining (Invitrogen)**,** while the apoptosis was evaluated using annexin V-FITC and 7-AAD (BD Biosciences), according to manufacturer’s instructions. **(D)** Viability of CLL cells transfected with STAT3-shRNA was not significantly impaired when compared with empty vector transfection and untreated cells.

**Supplementary Fig 3. Curcurbitacin and lenalidomide do not alter total STAT3 levels. CLL cells were** culture with 10 μM lenalidomide or 0.05 μM cucurbitacin for either 2 or 12 hours. The cells were then fixed, permeabilized and stained for total STAT3 (anti-human STAT3-PE antibody, Biolegend) (n=4).

**Supplementary Fig 4. IL-10 production by CLL cells and healthy control B cells** **after CXCL12 stimulation, (A).** CLL cells were stimulated with CpG (4 µg/ml) or CXCL12 (250 ng/ml) for 14-16 hours, followed by phorbol myristate acetate (PMA; 50 ng/mL) and ionomycin (250 ng/mL, Sigma Aldrich) for the last 6 hours of culture. IL-10 was measured in culture supernatants using IL-10 OptEIA ELISA kits (BD) according to the manufacturer’s instructions. (n=16). **(B)** CpG induces p-S727-STAT3 phosphorylation. CLL cells were stimulated with 4 μg/m CpG and evaluated for p-S727-STAT3 according to the phosflow protocol in materials and methods.

**(C)** CXCL12 does not induce IL-10 production by healthy control B cells. PBMC from healthy controls were exposed to 4 μg/ml of CpG or 250ng/ml CXCL12 for 8-10 hours, followed by addition of PMA, ionomycin and BFA (PIB), then incubated for another 6 hours before performing intracellular staining for IL-10 production. Cells are gated on CD19+ B cells.

**Supplementary Fig 5. CXCR4 expression on CLL cells positively correlates with CXCL12-induced IL-10+ CLL cells (%).** CLL cells were thawed and immediately stained for CXCR4 using a BV605 conjugated antibody (BioLegend). We gated on live CD19+CD5+ cells to measure CXCR4 expression on the surface of CLL B cells. CXCR4 expression is represented by the MFI index (n=20).

**Supplementary Fig 6. CXCL12 does not significantly alter healthy T cell function.** Negatively-selected T-cells from healthy controls were cultured either alone or with 250 ng/ml CXCL12 for 48 hours. The cells were then stimulated with CD3/CD28 magnetic beads (Invitrogen) for 6 hours followed by CD107a degranulation assay and intracellular staining for IFN-γ and TNF-α and IL-2.

**Supplementary Fig 7. CXCL12 stimulated CLL cells induce suppression of T cell proliferation.** CFSE labeled negatively-selected T-cells from 2 healthy donors were cultured either alone, with CLL cells or with 250 ng/ml CXCL12 stimulated CLL cells in a 1:10 ratio for 3 days (3 CLL donors) followed by surface staining and analysis.

**Supplementary Fig 8. Lenalidomide dose effect on CXCL12 induced S727-STAT3 phosphorylation as measured by Phosflow.** CLL cells were exposed to different concentrations of lenalidomide for 2 hours, followed by incubation with CXCL12 (250 μg/ml) for 20 minutes, fixed, permeabilized and stained for p-S727-STAT3.

**Supplementary Fig 9. Lenalidomide prevents CXCL12-induced phosphorylation of S727-STAT3 as measured by western blotting.** CLL cells were cultured with or without lenalidomide (10 μM) for 2 hours followed by incubation with CXCL12 (250 μg/ml) for 20 minutes. Cells were then lysed for western blotting and band thickness was measured and calculated as described in Materials and Methods. (n=4).

**Supplementary Fig 10. lenalidomide does not significantly alter healthy T cell function.** Negatively-selected T-cells from healthy controls were cultured either alone or with 10 μM lenalidomide for 48 hours. The cells were then stimulated with anti-CD3/CD28 magnetic beads (Invitrogen) for 6 hours followed by CD107a degranulation assay and intracellular staining for IFN-γ, TNF-α and IL-2 production.
